# Directed evolution on compact landing pads yields highly efficient recombinases for large DNA integration

**DOI:** 10.1093/nar/gkaf1444

**Published:** 2026-01-06

**Authors:** Hanseop Kim, Hyo-Gu Kang, Yeounsun Oh, Wi-jae Lee, Youngjeon Lee, Eun-Kyoung Kim, Ki-Hoan Nam, Dong-Seok Lee, Seung Hwan Lee, Bo-Woong Sim

**Affiliations:** National Primate Research Center, Korea Research Institute of Bioscience and Biotechnology, Cheongju 28116, Republic of Korea; Futuristic Animal Resource and Research Center, Korea Research Institute of Bioscience and Biotechnology, Cheongju 28116, Republic of Korea; Department of Life Science, Chung-Ang University, Seoul 06974, Republic of Korea; National Primate Research Center, Korea Research Institute of Bioscience and Biotechnology, Cheongju 28116, Republic of Korea; Department of Life Science, Chung-Ang University, Seoul 06974, Republic of Korea; National Primate Research Center, Korea Research Institute of Bioscience and Biotechnology, Cheongju 28116, Republic of Korea; KRIBB School of Bioscience, Korea National University of Science and Technology, Daejeon 34113, Republic of Korea; Laboratory Animal Resource and Research Center, Korea Research Institute of Bioscience and Biotechnology, Cheongju 28116, Republic of Korea; Laboratory Animal Resource and Research Center, Korea Research Institute of Bioscience and Biotechnology, Cheongju 28116, Republic of Korea; BK21 FOUR KNU Creative BioResearch Group, School of Life Sciences, Kyungpook National University, Daegu 41566, Republic of Korea; Department of Life Science, Chung-Ang University, Seoul 06974, Republic of Korea; Futuristic Animal Resource and Research Center, Korea Research Institute of Bioscience and Biotechnology, Cheongju 28116, Republic of Korea; KRIBB School of Bioscience, Korea National University of Science and Technology, Daejeon 34113, Republic of Korea

## Abstract

Site-specific DNA recombinases are powerful tools for genome engineering. The recent convergence of DNA recombinase-mediated technology with prime editing has established a promising new frontier for large-scale genomic integration. However, this strategy has limitations, including low intrinsic catalytic efficiency of DNA recombination enzymes and inefficiency of their long DNA landing pad insertions. To overcome these challenges, we developed a novel directed evolution strategy using progressively shortening landing pads as a selective pressure. The resulting evolved variants, VK and AVK, exhibited substantially enhanced intrinsic activity that is maintained on short-length landing pads. This enables a powerful synergistic effect with increased insertion efficiency of prime editing, leading to a dramatic increase in overall integration efficiency while maintaining high genomic specificity. This work thus provides a new class of highly efficient recombinases and a robust engineering strategy with broad applicability for both the development of gene therapies and fundamental research.

## Graphical Abstract

**Graphical Abstract:**
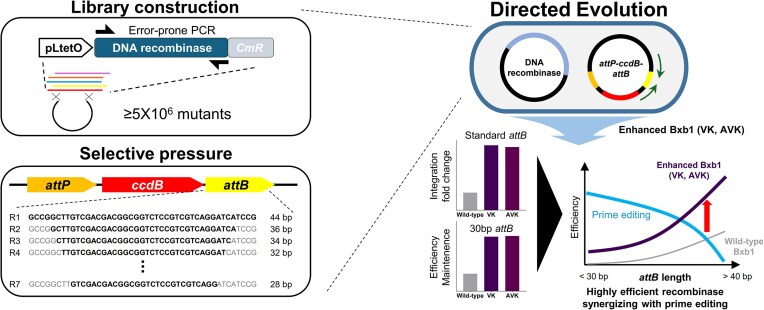

## Introduction

The advancement of genome editing technologies, such as recent CRISPR-based tools [1–5], has dramatically advanced the ability to enable more precise and efficient *in vivo* genome editing for *in vivo* genomes. However, despite their versatility, current CRISPR approaches still face significant challenges when attempting targeted genomic integration of large DNA sequences [6–11]. To overcome these constraints, site-specific DNA recombinase-based technologies have emerged as a compelling alternative, enabling precise and irreversible integration of large DNA cargoes into pre-inserted landing pad sequences such as *attP* and *attB* [12–16]. However, the practical application of these enzymes is often hindered by a fundamental limitation in their intrinsic recombination efficiency, which could result in modest integration rates even when landing pads are successfully installed [12, 15, 17, 18]. These findings suggest that fundamental improvements in the catalytic activity of recombination enzymes themselves could be a key solution for enhancing the efficiency of large-scale DNA genome editing.

The characteristics of recombinant enzymes that require long landing pads could often lead to an overall decrease in the efficiency of large-scale DNA genome editing. Methods such as homology-directed repair and prime editing (PE) offer the advantage of precisely inserting landing pads; however, they have the characteristic that insertion efficiency decreases significantly as the insertion size increases [19–21]. Even if insertion efficiency is increased by using a short landing pad, the characteristic of recombinases that efficiency decreases significantly as the landing pad becomes shorter could result in a decrease in overall integration efficiency, thereby nullifying the advantage of the short landing pad [13]. These characteristics suggest that alleviating the stringent length requirement (typically ≥38 bp) of recombinases such as Bxb1 to function properly [13, 16, 22] might help increase the fundamental efficiency of recombination enzymes and, in turn, improve overall integration efficiency.

The application of DNA recombinase-based technologies holds significant promise for fields such as the creation of transgenic animal models and gene therapies involving gene transfer [23–26]. The successful deployment of these technologies, however, hinges upon the efficiency and fidelity of the recombinase enzyme itself [27]. Several studies have demonstrated considerable efforts to engineer improved variants that overcome the limitations of wild-type (WT) enzymes based on methods such as phage-assisted directed evolution [28, 29]. These preceding endeavors highlight the critical need for novel engineering strategies that can robustly enhance recombinase performance.

In this study, we developed and characterized a suite of highly efficient Bxb1 variants, engineered through a novel directed evolution strategy that uses progressively shortening landing pads as a selective pressure. These variants exhibit substantially enhanced intrinsic activity on standard-length landing pads across diverse human genomic loci. Crucially, this enhanced intrinsic activity allows the variants to function efficiently on short landing pads, enabling a powerful synergistic effect with the increased insertion efficiency of PE. This combination overcomes both the intrinsic and delivery-related bottlenecks of previous systems, all while maintaining high genomic specificity. Collectively, this work establishes a robust and widely adaptable platform that augments both the efficiency and versatility of site-specific recombinase-mediated genome editing.

## Materials and methods

### Selection vector plasmid construction

The *attP*-pBAD-*ccdB*-*attB* selection vector was custom-synthesized by Vectorbuilder. In this construct, while the *attP* site was strategically positioned upstream of the pBAD promoter, the *attB* site was located downstream of the *ccdB* gene. To generate selection vectors with progressively shortened *attP* and *attB* sequences, this initial vector served as the backbone. Shorter *attP*/*attB* variants were constructed using specific primer sets (details in Supplementary Table S1) in conjunction with NEBuilder HiFi DNA Assembly Master Mix (New England Biolabs (NEB), Ipswich, MA, USA), following the manufacturer’s instructions.

### Bxb1 mutant library construction

The *Escherichia coli* codon-optimized Bxb1 gene was synthesized by Integrated DNA Technologies (IDT, Coralville, IA, USA). This gene was then cloned into a pLtetO-based vector (Addgene, Cambridge, MA, USA; plasmid #62655), enabling tetracycline-dependent expression. To generate a diversified Bxb1 mutant library, the entire coding sequence (CDS) of Bxb1 was subjected to error-prone polymerase chain reaction (PCR) using Genemorph II (Agilent, Santa Clara, CA, USA), aiming for a low mutation frequency of ~4–5 mutations per kilobase (used primer sets in Supplementary Table S1). The resulting PCR products (library inserts) were subsequently assembled into the pLtetO backbone using NEBuilder HiFi DNA Assembly Master Mix (NEB). The prepared library mixture was transformed into Endura electrocompetent cells (Lucigen (LGC), Middleton, WI, USA) via electroporation, performed using a Gene Pulser Xcell Total System (Bio-Rad, Hercules, CA, USA). Transformed cells were then plated on chloramphenicol (12.5 μg/ml) LB plates and incubated overnight at 37°C. A minimum of 5 × 10^6^ colonies were harvested, and plasmid DNA (library vector) was purified using a NucleoBond Xtra Maxi Plus kit (Macherey-Nagel (MN), Düren, Germany).

### Directed evolution and screening

For the directed evolution experiments, the *E. coli* strain BW25141 (obtained from NBRP, Japan) was utilized. First, BW25141 cells were transformed with the *attP*-pBAD-*ccdB*-*attB* selection vector and incubated overnight on ampicillin (50 μg/ml) LB plates. The transformed cells were then cultured until the OD_600_ reached 0.6, after which they were prepared as electrocompetent cells using a 10% glycerol solution. For each round of selection, 10 ng of the Bxb1 library vector was electroporated into the electrocompetent cells containing the selection vector, using the Gene Pulser Xcell Total System (Bio-Rad). Following electroporation, cells were incubated in 250 μl of SOC media for 30 min. A 1/100 aliquot of the incubated cells was plated on L-arabinose negative plates (containing ampicillin and chloramphenicol) to serve as a control and for calculating input colony counts. The remaining cells were split into two equal portions: half were plated on ampicillin/chloramphenicol/L-arabinose (4 mM) plates, and the other half on ampicillin/chloramphenicol/L-arabinose/aTc (0.1 μg/ml) plates. All plates were incubated overnight at 37°C. Input and output colony counts were determined using OpenCFU software [30]. Colonies from the L-arabinose/aTc positive plates were entirely harvested, and plasmid DNA (representing the library of that specific round) was purified using a NucleoBond Xtra Midi Plus kit (MN) for use in the subsequent selection round. This iterative process was continued by progressively shortening the *attP* and *attB* sequences in the selection vector. After all rounds were completed, Sanger sequencing was performed on individual colonies from rounds 6 and 7, which showed the largest differences in survival ratios, to analyze and identify the enriched mutations.

### Cell-lysate-based *in vitro* recombination assay

HEK293 cells were transfected with pCMV-Bxb1 variants linked to T2A-mEGFP. Seventy-two hours post-transfection, cells were harvested and lysed in Lysis Buffer [20 mM HEPES, pH 7.5, 100 mM KCl, 5 mM MgCl2, 5% glycerol, 1 mM dithiothreitol (DTT), 0.1%, Triton X-100, and 1× Halt Protease Inhibitor Cocktail (Thermo Scientific)]. The relative concentration of recombinase in each lysate was normalized based on mEGFP fluorescence intensity measured using a microplate reader. *In vitro* recombination reactions were performed in a 20 μl total volume containing 500 ng of *attP* plasmid (4.7 kb), *attB* PCR product (200 bp amplicon containing 38 or 30 bp core), and normalized cell lysates (1×, 0.5×, 0.25×) in reaction buffer (20 mM Tris–HCl, pH 7.5, 10 mM ethylenediaminetetraacetic acid, 100 mM KCl, 50 mM NaCl, 5 mM MgCl_2_, 1 mM spermidine, 1 mM DTT, 100 µg/ml Salmon Sperm DNA, and 5% glycerol). The molar ratio of *attB* to *attP* was adjusted to 4:1 for the 38 bp *attB* substrate and 10:1 for the 30 bp *attB* substrate. Reactions were incubated at 30°C for 1 h. Following incubation, DNA was purified using HiGene Gel & PCR Purification System (BIOFACT, Daejeon, Republic of Korea, GP104) and analyzed by electrophoresis on a 0.8% agarose gel. To quantify recombination activity, the band intensities of the linear recombination product (∼4.9 kb) and the remaining supercoiled substrate were measured using ImageJ software (NIH).

### Mammalian cell expression vector construction

Human codon-optimized DNA sequences for pCMV-Bxb1 WT, VK, AVK, evo, ee, and c2 variants were either directly synthesized or subcloned into the pCMV backbone. The corresponding amino acid sequences and DNA sequences are described in detail in the Supplementary Information. For the *in vitro* recombination assay, we constructed expression vectors encoding Bxb1 variants linked to EGFP via a T2A self-cleaving peptide (Bxb1-T2A-EGFP) to allow for protein quantification. The prime editor used was pCMV-PEmax (Addgene plasmid #174820). PE6d and PE7 expression vectors were constructed by cloning the corresponding prime editor architectures into the pCMV-PEmax backbone. For guide RNA expression, pU6-based SpCas9 guide RNA backbone vector (Addgene plasmid #132778) was utilized for both pegRNAs and nicking sgRNAs. The *attP* cargo vector was constructed by inserting the 52 bp *attP* sequence into an mEGFP-N1 backbone (Addgene plasmid #54767) (Supplementary Table S2). Mutation shuffling for generating specific variant combinations was performed through Gibson assembly (NEBuilder HiFi DNA Assembly Master Mix, NEB), using primer sets designed to incorporate desired mutant amino acid residues (Supplementary Table S3).

### Cell culture and transfection

HEK293 and NIH 3T3 cells were used for mammalian cell experiments. Cells were maintained in Dulbecco’s modified Eagle medium (Gibco, Grand Island, NY, USA) supplemented with 10% fetal bovine serum (Gibco) and incubated at 37°C with 5% CO_2_. For PE to insert *attB* sequences into target genomic DNA, cells were subjected to electroporation using a NEPA21 system (NEPAGENE, Ichikawa, Japan), strictly following the manufacturer’s protocol. To generate the pre-*attB* inserted cell populations used in the experiments for a standard-length 38 bp *attB* site was inserted into target genomic loci. This was achieved using the twin PE method, which utilizes a pair of pegRNAs. Briefly, 1 × 10^6^ cells were electroporated with 5 μg of pCMV-PEmax and 1 μg each of the two corresponding pegRNAs. For the experiments in Figs 4 and 5D, *attB* sites were inserted using the PE3 method; 2 μg of pegRNA and 0.1 μg of nicking sgRNA. Following electroporation, cells were seeded into six-well plates and cultured for 96 h. Subsequently, for site-specific integration assays, the prime-edited cells were re-seeded at a density of 1 × 10^5^ cells per well in 24-well plates. After 16–24 h, when cell confluency reached 60%–70%, recombinase expression plasmids and the *attP* cargo vector were delivered via Lipofectamine 3000 (Thermo Fisher Scientific, Carlsbad, CA, USA). For each well, a transfection mixture was prepared by combining 300 ng of pCMV-Bxb1 (WT or mutant variants), 300 ng of pCargo-mEGFP (*attP*), 1 μl of P3000 reagent, and 1.8 μl of Lipofectamine 3000 reagent in 50 μl of Opti-MEM (Gibco). The mixture was incubated for 10 min at room temperature before being added to the cells. Cells were harvested 72 h post-transfection, and genomic DNA was extracted and purified using a genomic DNA prep kit (Bioneer, Daejeon, Republic of Korea, K-3032) according to the manufacturer’s instructions. Mouse embryonic stem cell (mESC) (ATCC, Manassas, VA, USA, SCRC-1002) Culture and Transfection were maintained on gelatin-coated dishes in 3i/LIF medium: KnockOut DMEM (Gibco) supplemented with 10% KnockOut Serum Replacement (Gibco), 1% GlutaMAX (Gibco), 1% non-essential amino acids (Gibco), 0.1 mM β-mercaptoethanol (Gibco), 1000 U/ml LIF (PeproTech, Gibco), 0.8 μM PD184352, 2 μM SU5402, and 3 μM CHIR99021. For transfection, 1 × 10^6^ cells were electroporated with electroporated with 5 μg of pCMV-PEmax and 1 μg each of the two corresponding pegRNAs using a NEPA21 system (NEPAGENE, Ichikawa, Japan) according to the manufacturer’s optimized protocol (105 V, 5 ms, 2 pulses). Following electroporation, cells were seeded onto mitomycin C-treated mouse embryonic fibroblast feeder layers in six-well plates and cultured for 72 h. Subsequently, for site-specific integration assays, the prime-edited cells were re-seeded onto 0.1% gelatin-coated plates under feeder-free conditions at a density of 1 × 10⁵ cells per well in 24-well plates. After 16 h, when cell confluency reached 60%–70%, recombinase expression plasmids and the *attP* cargo vector were delivered via Lipofectamine 2000 (Thermo Fisher Scientific, Carlsbad, CA, USA), following the manufacturer’s protocol. For each well, a transfection mixture was prepared by combining 300 ng of pCMV-Bxb1 (WT or mutant variants), 300 ng of pCargo-mEGFP (*attP*), and 2 μl of Lipofectamine 2000 reagent in 50 μl of Opti-MEM (Gibco). The mixture was incubated for 20 min at room temperature before being added to the cells. Cells were harvested 72 h post-transfection, and genomic DNA was extracted as described above.

### Targeted amplicon sequencing (NGS) and analysis

To quantify *attB* insertion efficiency resulting from PE and to assess site-specific integration efficiency, targeted deep sequencing was performed. Amplicon libraries from extracted genomic DNA were prepared through a two-step PCR process, utilizing KOD One polymerase (Toyobo, Osaka, Japan) for both steps. Next-generation sequencing (NGS) was carried out on an Illumina Mini-Seq system (Illumina, San Diego, CA, USA; SY-420-1001). For the quantification of site-specific integration events, a three-primer NGS assay, adapted from *Yarnall et al*., was employed. This assay is composed of a pair of adapter primers binding to the target genomic locus and a third adapter primer binding near the *attP* site within the cargo vector. The first PCR step included 100–200 ng of genomic DNA, 0.4 μM each of a target forward primer and a cargo forward primer, and 0.8 μM of a target reverse primer in a total 5 μl reaction mix containing KOD One polymerase (Toyobo). This reaction was performed for 18 cycles according to the manufacturer’s instructions. Subsequently, 1/20 of the diluted PCR product from the first step was used as template for the second PCR, which incorporated index primers (0.8 μM each) in a total 15 μl mixture and was run for 20 cycles under identical conditions. A 10 μl aliquot of the final PCR product was purified using a HiGene Gel & PCR Purification System (BIOFACT, Daejeon, Republic of Korea, GP104) and quantified for NGS. Sequencing read fastq files were analyzed using Cas-Analyzer within CRISPR RGEN Tools (http://www.rgenome.net) [31]. The indel frequency ratio, including *attB* insertion within the targeted genomic locus, was calculated as (precise insertion reads / targeted genomic locus reads) * 100. The integration ratio was calculated as (targeted integration reads / (target genomic locus reads + targeted integration reads)) * 100. For off-target analysis, pseudo-*attB* sites were selected based on methodologies described by *Bessen et al*. and *Yarnall et al*. The specific sequences of these pseudo-*attB* sites are indicated in Fig. 6A (right panel). Off-target integration ratios were calculated using the same formula as for on-target integration ratios. All primer information for off-target analysis is detailed in Supplementary Table S4.

## Results

### Directed evolution identifies Bxb1 recombinase variants with improved activity on shortened *attB* sequences

**Figure 1.**
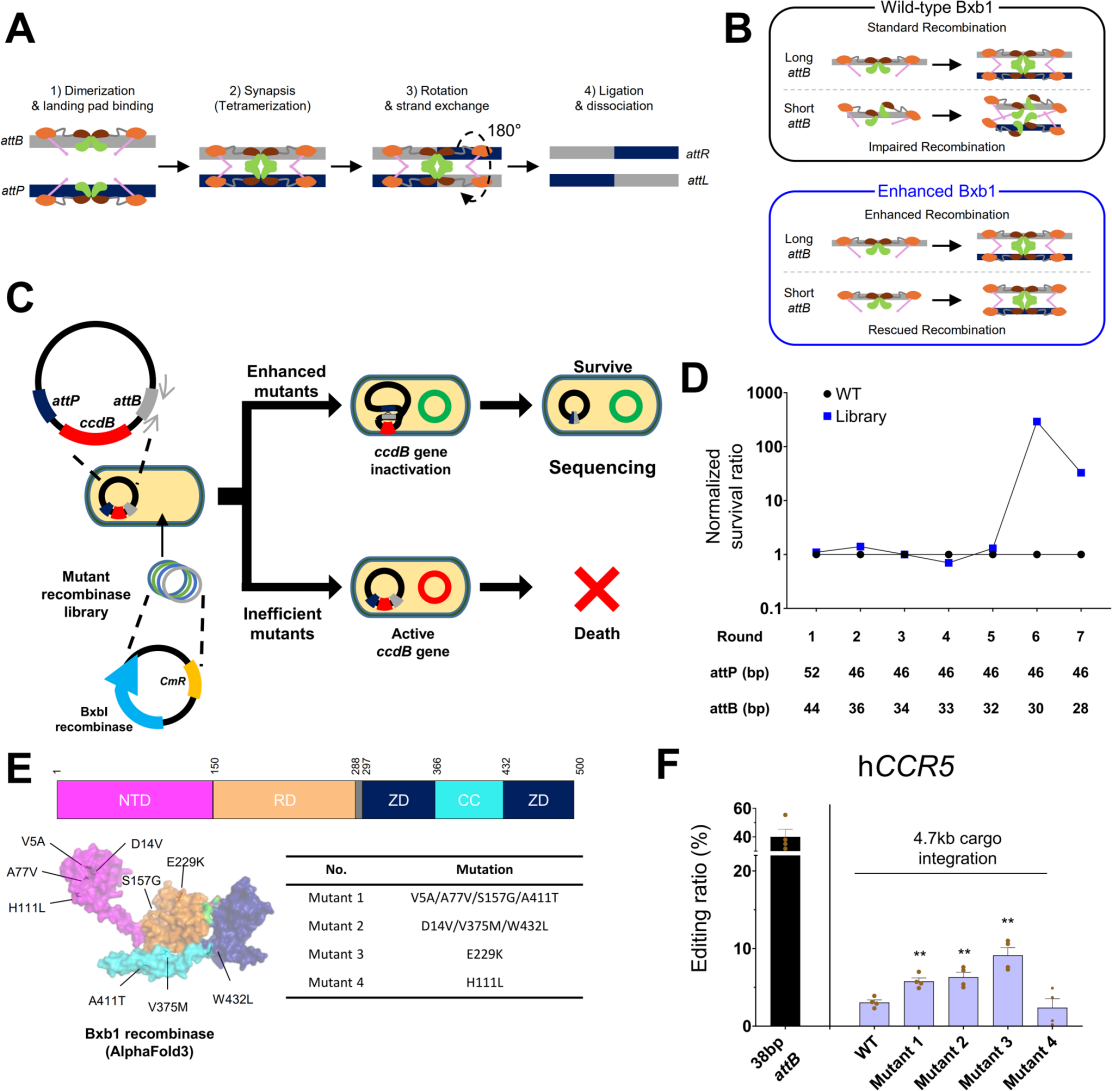
Directed evolution strategy and initial validation of enhanced Bxb1 variants. (**A**) A schematic depicting the four-step mechanism of serine recombinase-mediated DNA exchange: (i) recombinase dimers bind to attachment sites (*attB* and *attP*); (ii) formation of a tetrameric synaptic complex; (iii) 180° rotation and strand exchange; and (iv) ligation and dissociation to yield *attL* and *attR* products. (**B**) Conceptual model illustrating the proposed mechanism of action on shortened *attB* landing pads. While WT Bxb1 performs standard recombination on long *attB* sites, it exhibits impaired recombination on short sites, likely due to defects in productive synaptic complex assembly. In contrast, the enhanced variants are hypothesized to possess improved intrinsic properties that enable rescued recombination on short sites, and enhanced recombination on long sites by facilitating the formation of a productive synaptic complex. (**C**) Schematic of the *E. coli*-based directed evolution system. A library of recombinase mutants was screened for the ability to excise a toxic controller of cell death or division B (*ccdB*) gene flanked by landing pads, linking survival to recombination efficiency. Enhanced mutants excise the *ccdB* gene, leading to host survival, while inefficient mutants do not, resulting in host cell death. (**D**) Normalized survival ratio of the Bxb1 variant library relative to WT over seven rounds of selection with progressively shortened landing pads. The ratio was calculated from survived colonies post-induction, normalized to the electroporation input. (**E**) A summary of the nine recurrent amino acid substitutions identified from the screen. The table lists the mutations found in four representative mutants. The domain architecture of Bxb1 and the locations of the mutations are mapped onto a structure predicted by AlphaFold3 [NTD (N-terminal catalytic domain, 1–150), RD (recombinase domain, 151–288), ZD (zinc ribbon domain, 297–366, 433–500), and CC (coiled-coil domain, 367–432)]. (**F**) The cargo integration efficiency of WT Bxb1 and four representative mutant variants in HEK293 cells with a pre-inserted 38 bp *attB* site. Data are represented as the mean of *n* = 4 independent experiments; error bars, SEM. *P* values for comparisons to WT were determined by one-way analysis of variance (ANOVA). Comparisons not marked with an asterisk are not statistically significant (*P < .05; **P < .01; ***P < .001).

Site-specific recombination by serine recombinases is a multi-step process, wherein dimers of the recombinase bind to the *attP* and *attB* sites. These protein–DNA complexes then associate to form a tetrameric synaptic complex, which mediates strand exchange through subunit rotation to generate the final *attL* and *attR* product sites (Fig. 1A). The efficiency of this process is critically dependent on the stability of the synaptic complex. Previous work has established that the distal “arm” regions of the *attB* attachment site are crucial for synaptic complex assembly; truncating or mutating these arms is known to compromise synaptic competence and reduce recombination activity [22, 32–35]. We therefore designed our directed evolution strategy based on the rationale that using a shortened landing pad as a selective pressure would enrich for variants with enhanced intrinsic recombinase activity and robust substrate tolerance. We hypothesized that, while WT Bxb1 would exhibit impaired synaptic function on a shortened *attB* pad, an “enhanced” variant selected under this pressure would evolve to maintain robust activity. This enhanced intrinsic efficiency would not only rescue recombination on short pads but would also lead to superior performance on standard-length pads, owing to a fundamentally improved enzymatic mechanism (Fig. 1B).

To generate such variants, we established an *E. coli*-based bacterial selection platform that links Bxb1 recombinase activity to survival (Fig. 1C, detailed in Supplementary Fig. S1) [36, 37]. In this system, a toxic *ccdB* gene, flanked by *attP* and *attB* sequences, was placed under the control of an arabinose-inducible pBAD promoter. The expression of Bxb1 variants was regulated by an anhydrotetracycline (aTc)-inducible pLtetO-based plasmid. Effective recombination between *attP* and *attB* sites resulted in the precise excision of the *ccdB* cassette, enabling bacterial survival in the presence of arabinose induction (Supplementary Fig. S2). Variants lacking sufficient recombination activity failed to excise the cassette, resulting in cell death from *ccdB*-mediated toxicity.

A library of Bxb1 variants was generated through error-prone PCR and screened over seven iterative rounds of selection. During the selection process, the *attB* and *attP* sequences were progressively shortened to increase selection pressure. To quantify the degree of enrichment, we measured the survival rate in each round and normalized the survival rate of the variant library to that of WT Bxb1 under the same conditions (Fig. 1D). Through this normalization, we were able to directly compare the relative degree of enrichment of active variants across the selection rounds. In rounds 1 through 5, where the *attB* site was gradually shortened from 46 to 32 bp and *attP* was adjusted accordingly (Supplementary Table S1), the normalized survival of the variant library remained close to 1, indicating that the selection pressure was within the range of WT activity. However, in round 6, where the *attB* site was shortened to 30 bp, the WT Bxb1 group showed a sharp decrease in absolute survival (Fig. 1D and Supplementary Fig. S3), while the variant library maintained a relatively stable survival rate. As a result, the normalized survival of the variant library increased dramatically, reaching >200-fold higher than WT Bxb1 (Fig. 1D). In round 7, with the *attB* further reduced to 28 bp, the normalized survival of the variant library also declined, and the gap between WT and the library narrowed. This suggests that the applied selection pressure approached the activity limits of even the most enriched variants. To rigorously assess whether the identified variants confer a clonal-level survival advantage, we conducted individual *E. coli* survival assays on four representative mutants. Consistent with the library screening results, these mutants exhibited robust survival and distinct colony formation on shortened *attB* sites compared to WT (Supplementary Table S5 and  Supplementary Fig. S3). Notably, Mutant 4 exhibited a striking enhancement in survival, exceeding that of the WT by >460-fold in Round 6 and thereby substantiating its pronounced enrichment observed in the sequencing analysis. Sequencing analysis of surviving clones identified nine recurrent amino acid substitutions: V5A, D14V, A77V, H111L, S157G, E229K, V375M, A411T, and W432L (Fig. 1E and Supplementary Table S5).

To confirm whether our directed evolution strategy using a short landing pad as a selective pressure had indeed led to a global enhancement in variant efficiency, we performed an initial validation of representative variants in human-derived cells. We evaluated the ability of four variants from the bacterial screen to mediate site-specific integration of a 4.3 kb *attP* cargo vector in HEK293 cells containing a pre-inserted, 38 bp standard length *attB* site at the *CCR5* locus. Integration efficiency was quantified using a three-primer NGS assay [13]. Three of the four variants—Mutant 1 (V5A/A77V/S157G/A411T), Mutant 2 (D14V/V375M/W432L), and Mutant 3 (E229K)—showed modest but consistently improved integration efficiency compared to WT Bxb1 (Fig. 1F). Intriguingly, Mutant 4 (H111L), despite exhibiting the highest survival rate and enrichment in the bacterial screen, showed a reduction in efficiency in the mammalian context. These findings provided the first direct evidence that our selection strategy successfully enriched for variants with a fundamentally enhanced recombination activity in a mammalian context, while also highlighting the functional heterogeneity among the enriched clones.

### Functional evaluation of rationally shuffled Bxb1 variants in human-derived cells

**Figure 2.**
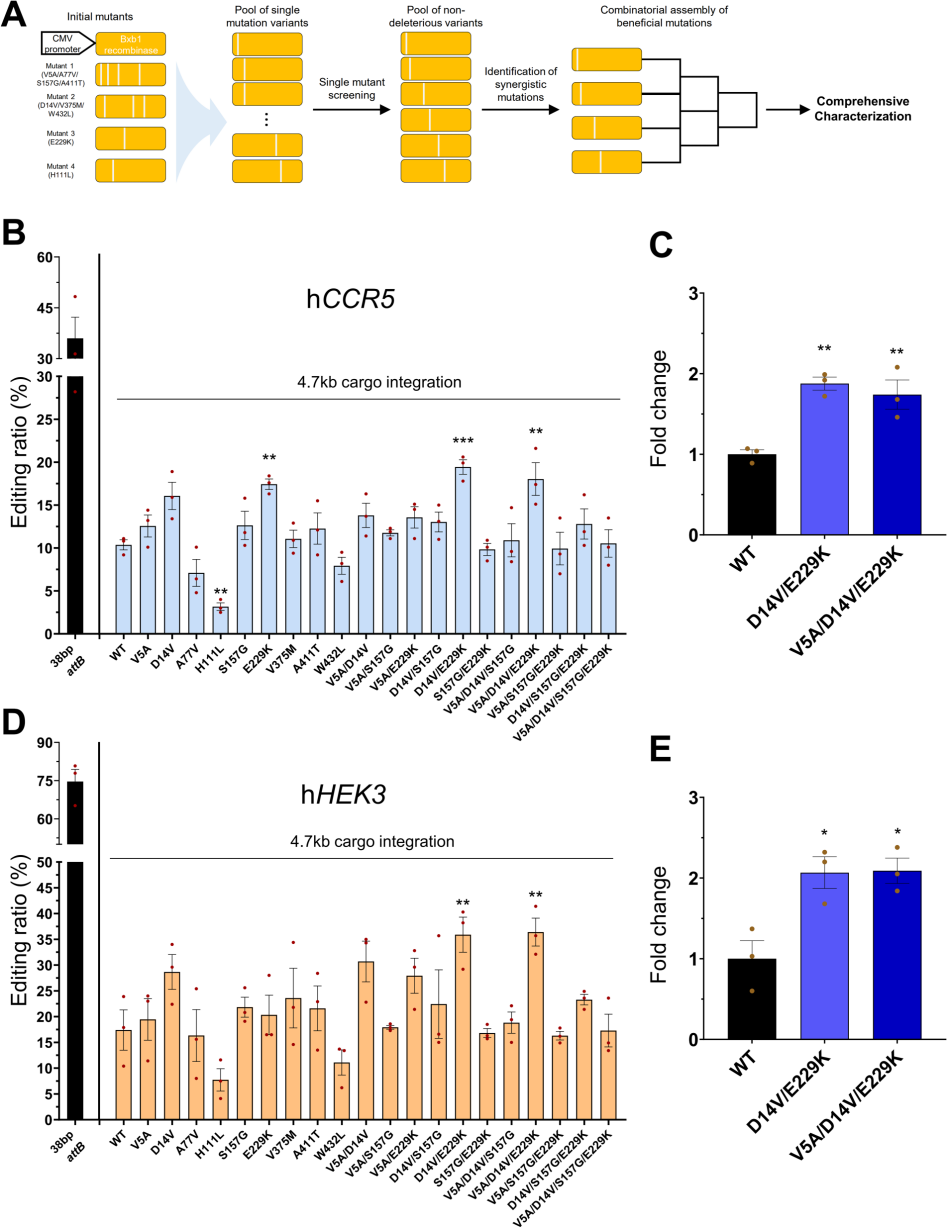
Rational shuffling of mutations identifies high-performance Bxb1 variants with enhanced activity in human-derived cells. (**A**) A schematic depicting the rational, multi-tiered strategy for generating the 20-variant library characterized in this study. The nine recurrent mutations were first screened as single mutants. Four key beneficial mutations (V5A, D14V, S157G, and E229K) were identified and used as building blocks to assemble double, triple, and quadruple mutants. The integration efficiencies of the 20 rationally selected variants at the (**B**) *CCR5* and (**D**) *HEK3* loci in HEK293 cells. The variants tested comprise the single, double, triple, and quadruple mutants generated in panel (A). For each locus, the efficiency of the pre-inserted 38 bp *attB* landing pad is shown for context (black), alongside the overall integration efficiency for each variant (colored). (**C, E**) The fold-change in integration efficiency for the top-performing variants identified in panels (B) and (D)—D14V/E229K (VK) and V5A/D14V/E229K (AVK)—relative to WT at the (C) *CCR5* and (E) *HEK3* loci. The mean efficiency of WT was normalized to 1.0. Data are represented as the mean of *n* = 3 independent biological replicates (dots); error bars, SEM. *P* values for comparisons to WT were determined by one-way ANOVA. Comparisons not marked with an asterisk are not statistically significant (*P < .05, **P < .01, ***P < .001).

Our initial screening in human-derived cells confirmed that variants from the directed evolution exhibit enhanced activity (Fig. 1F). To pinpoint the most critical amino acid substitutions and create even more potent recombinases, we implemented a rational, combinatorial strategy to further evaluate the nine recurrent mutations (Fig. 2A). We first characterized all nine as single-mutation variants to assess their individual effects. Consistent with our initial validation, the H111L substitution alone resulted in reduced activity compared to WT (Fig. 2B and D). In contrast, mutations such as V5A, D14V, S157G, and E229K consistently demonstrated enhanced integration efficiency. Based on this initial screen, we identified four key beneficial mutations—V5A, D14V, S157G, and E229K—that were selected as building blocks for further optimization. These four were then rationally shuffled to generate a focused library of synergistic double, triple, and quadruple mutants.

To conduct an unbiased assessment of the intrinsic activity of the resulting 20 variants, we tested them in HEK293 cells containing a pre-inserted, standard-length 38 bp *attB* site at two independent genomic loci, *CCR5* and *HEK3*. Upon evaluating all variants, a clear range of activities was observed (Fig. 2B and D). This screen identified the double mutant D14V/E229K (hereinafter VK) and the triple mutant V5A/D14V/E229K (hereinafter AVK) as the most potent variants. When compared to WT Bxb1, these top-performing variants demonstrated robustly enhanced activity, showing an average of ~1.9-fold higher efficiency at the *CCR5* site (Fig. 2C) and a more pronounced increase of ~2.1-fold at the *HEK3* site (Fig. 2E). Based on these findings, we next investigated whether VK and AVK maintain their enhanced efficiency across a broader range of genomic loci.

### Broadly enhanced activity of Bxb1 variants across diverse genomic loci in human-derived cells

**Figure 3.**
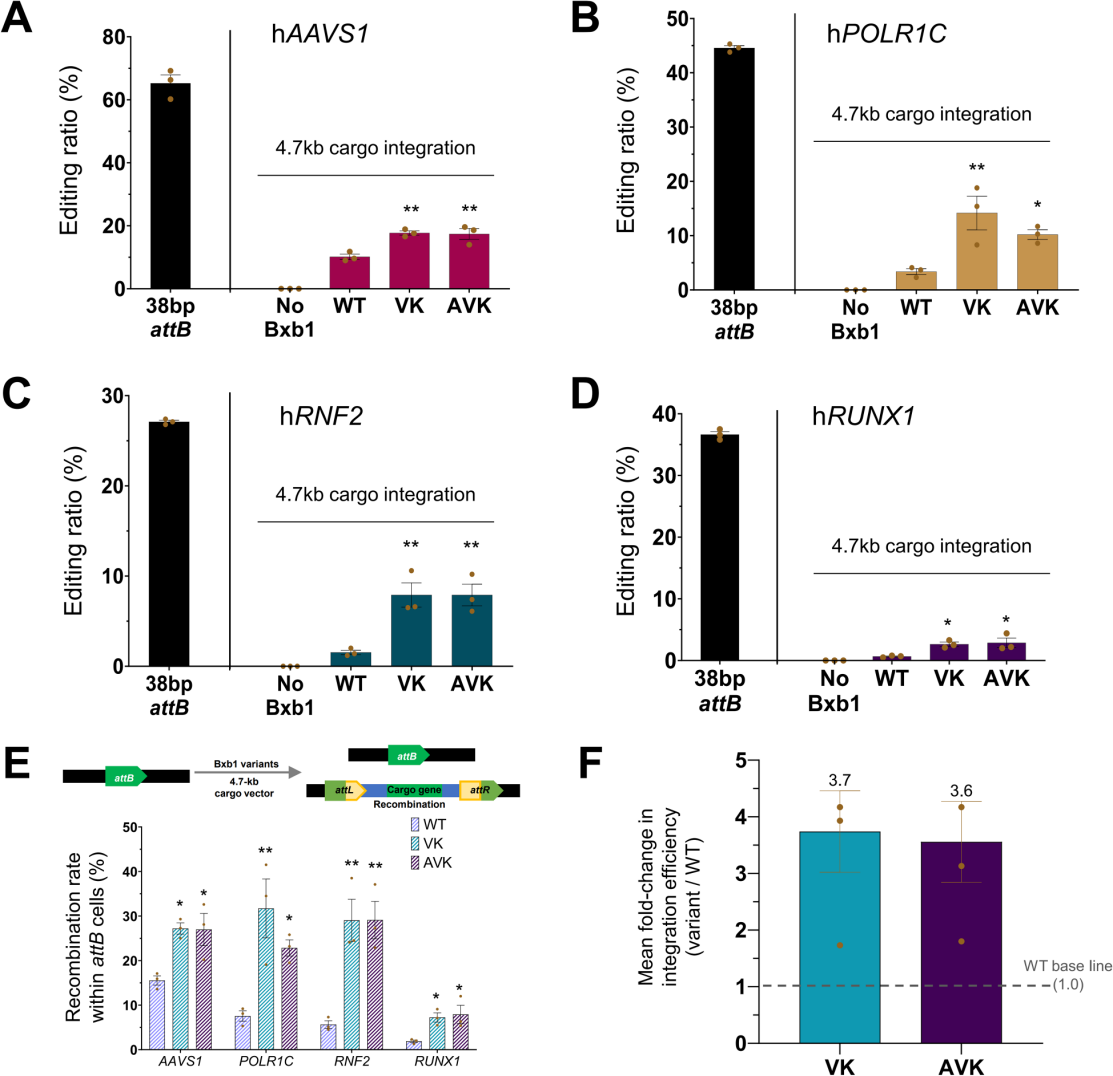
Broad enhancement of Bxb1 variant activity across multiple genomic loci. Comparison of *attB* landing pad insertion efficiency and subsequent overall integration efficiency for WT, VK, and AVK recombinases at four diverse genomic loci: *AAVS1* (**A**), *POLR1C* (**B**), *RNF2* (**C**), and *RUNX1* (**D**). Thirty-eight base pairs *attB* landing pad insertion efficiency (black) and the subsequent overall integration efficiency for WT, VK, and AVK recombinases (colored). (**E**) Recombination efficiency of Bxb1 variants per genomic locus, based on the percentage of *attB*-inserted cells. The intrinsic recombination efficiency of the variants, calculated by normalizing the overall integration efficiency to the *attB* insertion efficiency at each locus. (**F**) Average fold-change in integration efficiency of engineered variants relative to WT Bxb1 (WT) across all target gene loci. The mean efficiency of WT was normalized to 1.0. Data are represented as the mean of *n* = 3 independent biological replicates (dots); error bars, SEM. *P* values for comparisons to WT were determined by one-way ANOVA. Comparisons not marked with an asterisk are not statistically significant (*P < .05, **P < .01, ***P < .001).

To determine whether the enhanced efficiency of VK and AVK was broadly applicable, we evaluated their efficiency across four additional human genomic loci: *AAVS1, POLR1C, RNF2*, and *RUNX1*. For each site, we measured both the initial *attB* insertion ratio and the subsequent integration efficiency for WT, VK, and AVK in cells where a standard-length 38 bp *attB* sequence was pre-inserted (Fig. 3A–D). Across the four target sites, WT Bxb1 showed a relatively modest efficiency ranging from 0.7% to 10.1%, whereas the VK and AVK variants achieved significantly higher rates, reaching up to 19.0% and 19.1%, respectively.

To account for the variability in *attB* insertion and to assess the variants' intrinsic recombination activity, we normalized the integration rates to the *attB* insertion efficiency at each locus. This analysis revealed that the normalized efficiency for WT Bxb1 ranged from 1.9% to 15.5%, whereas VK and AVK achieved much higher efficiencies of 7.2%–31.7% and 5.4%–32.6%, respectively (Fig. 3E). This confirmed that both variants consistently outperformed the WT Bxb1 across all four sites. To summarize the overall efficiency gain, we calculated the average fold change in integration efficiency relative to WT. VK and AVK demonstrated substantial and reproducible improvements, with mean fold-changes of 3.7-fold and 3.6-fold, respectively (Fig. 3F).

### Engineered Bxb1 variants facilitate robust large DNA integration by exploiting the improved prime editing efficiency enabled through recognition of compact *attB* sequences

To comprehensively evaluate the utility of our engineered Bxb1 recombinase variants for working on short *attB* sites within the mammalian cell genome, we next assessed their integration efficiency at *attB* sites generated by traditional PE (PE3) [13]. This approach allowed us to investigate in detail the PE3 insertion efficiency according to *attB* length, overall cargo integration efficiency, and pure recombination efficiency of the recombinase variants. To minimize confounding factors arising from variable *attB* insertion efficiency and ensuring robust downstream integration analysis, we selected three genomic loci (*ACTB, NOLC1*, and *LNMB1*) previously characterized for high PE3 editing efficiency [13].

**Figure 4.**
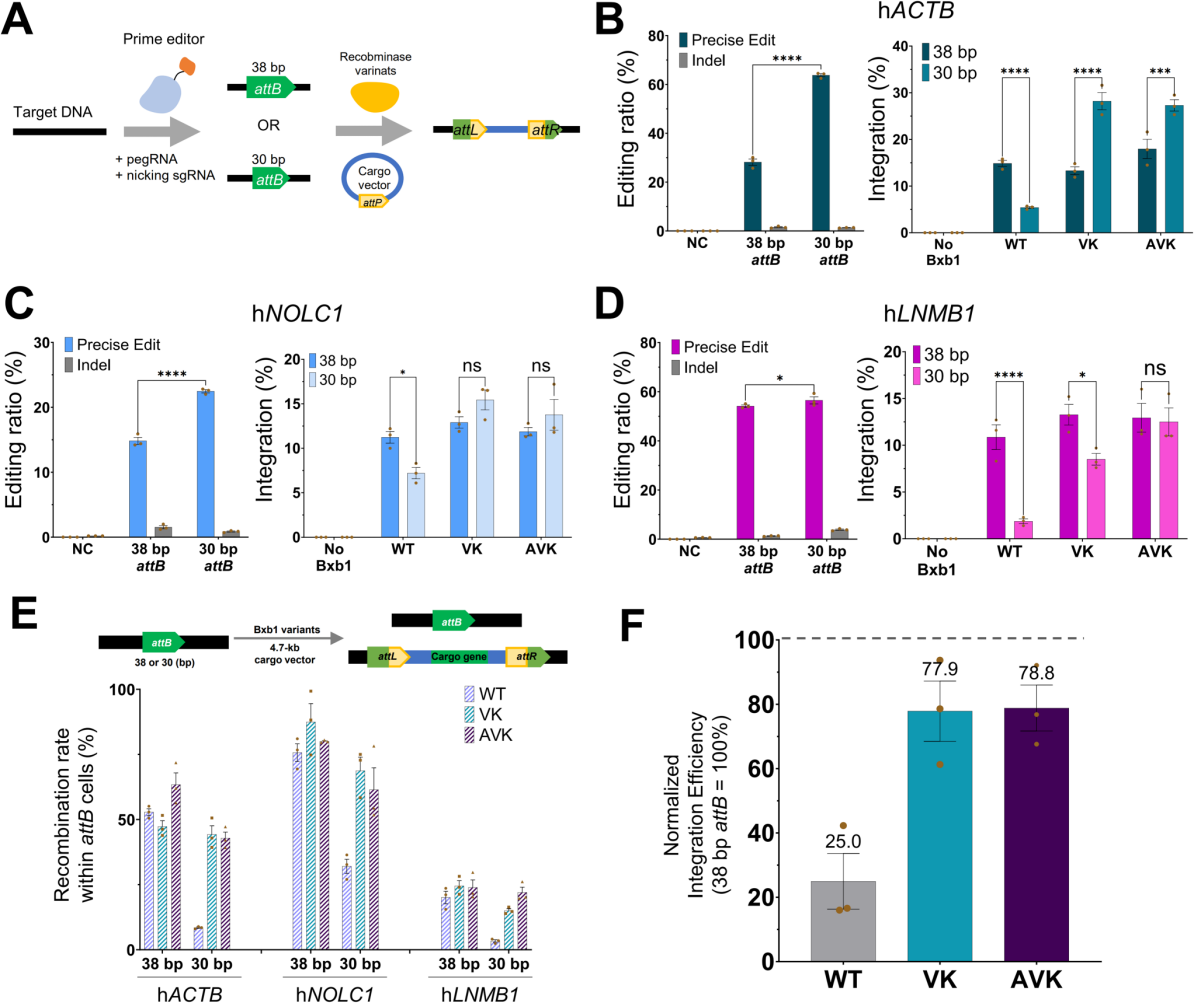
Engineered Bxb1 variants enhance overall gene integration by leveraging improved PE efficiency at short *attB* sites. (**A**) A schematic diagram of PE3-mediated *attB* landing pad insertion and subsequent Bxb1 recombinase-mediated cargo integration. PE3 efficiency for 38 bp versus 30 bp *attB* insertion (left panels) and integration efficiency of WT, VK, and AVK recombinases into 38 and 30 bp *attB* sites (right panels) at diverse genomic loci [*ACTB* (**B**), *NOLC1* (**C**), and *LNMB1* (**D**)]. (**E**) Recombination efficiency of Bxb1 variants in *attB*-inserted cells, comparing 30 bp versus 38 bp *attB* sites. The intrinsic activity of the recombinase, calculated by normalizing the overall integration efficiency to the *attB* insertion efficiency. (**F**) Relative change in integration efficiency of Bxb1 variants when transitioning from 38 to 30 bp *attB* sites. The average integration-efficiency preservation of WT, VK, and AVK when transitioning from a 38- to 30-bp *attB*, with the 38 bp efficiency set as 100% for each variant across the tested loci. Data are represented as the mean of *n* = 3 independent biological replicates (dots); error bars, SEM. *P* values for comparisons to WT were determined by one-way ANOVA. Comparisons not marked with an asterisk are not statistically significant (*P < .05, **P < .01, ***P < .001).

We used PE3 to insert a 38- or 30-bp *attB* sequence into the target genomic loci. Subsequently, a cargo vector containing an *attP* sequence was introduced along with various Bxb1 recombinase variants to induce site-specific integration (Fig. 4A). First, we assessed the efficiency of PE3 in inserting the 38-and 30-bp *attB* sequences at each of the three target loci (Fig. 4B–D, left panels). As anticipated for PE3 [13], which typically exhibits reduced efficiency with longer inserts, we observed a consistent and significant improvement in insertion efficiency when using the shorter 30 bp *attB* sequence compared to the 38 bp *attB* sequence across all tested sites. Specifically, at the *ACTB* locus (Fig. 4B, left panel), 30 bp *attB* insertion achieved 63.8% efficiency, a substantial increase from 28.2% for 38 bp. Similarly, at *NOLC1* (Fig. 4C, left panel), efficiency rose from 14.8% to 22.5% with the shorter *attB*. Even at *LNMB1* (Fig. 4D, left panel), which showed a relatively high baseline efficiency, a slight improvement was observed from 54.1% (38 bp) to 56.5% (30 bp), confirming the general trend of enhanced PE3 efficiency with shorter *attB* targets. These results unequivocally confirm that utilizing a shorter *attB* sequence significantly enhances the initial PE efficiency, thereby increasing the number of high-fidelity landing pads for subsequent site-specific recombination.

Next, we evaluated the integration efficiency of WT Bxb1, VK, and AVK variants into these PE3-generated *attB* sites of varying lengths (Fig. 4B–D, right panels). At the 38 bp landing pads, the efficiency enhancement of VK and AVK over WT was modest in these three genomic contexts, suggesting that locus-dependent effects could influence the observable efficiency gains. However, a clear and critical distinction in recombinase efficiency emerged at the 30 bp sites. For WT Bxb1, despite the higher initial availability of the 30 bp *attB* sequence, its subsequent integration efficiency showed a consistent, large reduction across all three loci. At the *ACTB* locus (Fig. 4B, right panel), WT integration efficiency dropped significantly from 14.9% to 5.4%, representing an ~2.7-fold reduction. Similarly, at *NOLC1* (Fig. 4C, right panel), it went from 11.2% to 7.2%, and at *LNMB1* (Fig. 4D, right panel), WT efficiency decreased sharply from 10.9% to 1.9%, consistently illustrating its diminished activity on shortened *attB* sequences despite increased landing pad availability. This consistent decline shows a fundamental and critical limitation of WT Bxb1, in that the benefit gained from increased PE efficiency with short *attB* sequences is mostly lost.

In stark contrast, our engineered variants, VK and AVK, demonstrated superior and more adaptable efficiency. When integrating into the 30 bp *attB* sites, their integration efficiencies were either minimally affected or, remarkably, showed an increase in efficiency compared to their efficiency at 38 bp *attB* sites. At *ACTB* (Fig. 4B, right panel), VK efficiency impressively increased from 13.3% to 28.2% (~2.1-fold increase), and AVK from 18.0% to 27.3% (~1.5-fold increase). Similarly, at *NOLC1* (Fig. 4C, right panel), VK also showed an increase from 12.9% to 15.4%, and AVK from 11.9% to 13.8%. Even at *LNMB1* (Fig. 4D, right panel), where WT showed a dramatic drop, VK maintained comparable efficiencies (13.3% to 8.5%), and AVK showed a more modest decrease (12.9% to 12.5%), demonstrating their ability to sustain activity on shorter *attB* despite the minimal PE3 efficiency gain. This crucial finding shows that VK and AVK effectively overcome the length-dependent activity reduction observed in WT Bxb1 while being able to maximize the benefits of PE enhanced by the short *attB* sequence.

To further delineate the intrinsic activity of the recombinase variants, we analyzed their pure recombination efficiency within the population of cells where *attB* sequences were successfully inserted via PE (Fig. 4E). This analysis, which normalizes for differences in initial landing pad delivery, allows for a clear comparison of the variants’ activity by *attB* length. It revealed that while WT recombinase showed a significant reduction in its recombination efficiency on 30 bp *attB* sites compared to 38 bp sites, VK and AVK largely preserved their high activity. This differential efficiency is clearly illustrated when comparing the average recombination efficiency preservation from 38 to 30 bp *attB* sites across the tested loci (Fig. 4F). While WT recombinase exhibited a significant reduction in efficiency, dropping to an average of 25.0% relative to its 38 bp efficiency, our engineered variants demonstrated remarkable retention of activity. VK maintained 77.9% and AVK retained 78.8% of their 38 bp efficiency, showcasing their robust ability to sustain activity on shortened landing pads.

### Elucidation of the mechanisms underlying the enhanced activity of engineered Bxb1 recombinases and their application in biologically relevant cellular system

**Figure 5.**
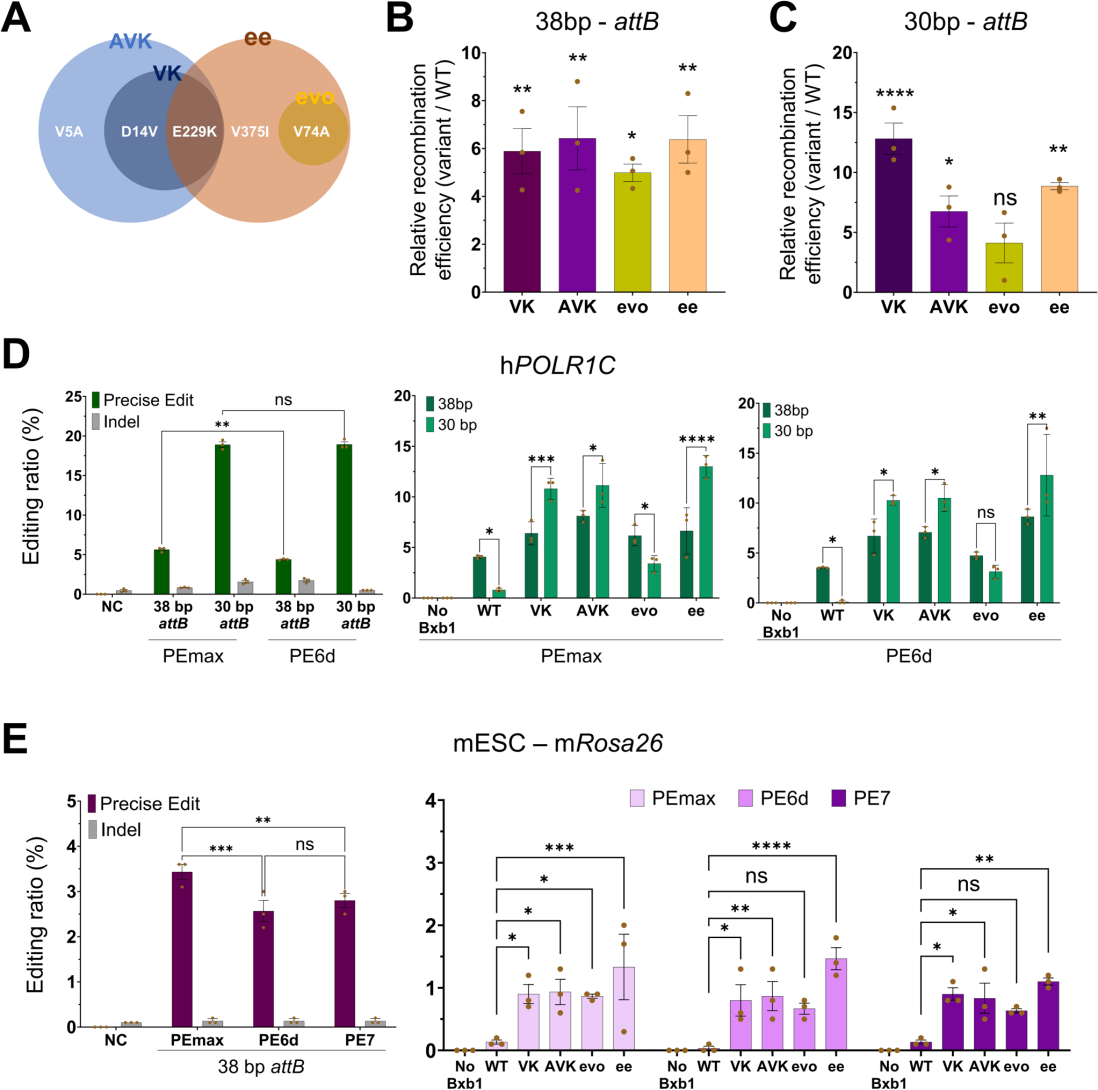
A comparative evaluation of engineered Bxb1 recombinase variants across multiple cell lines and physiologically relevant cellular systems. (**A**) Genotypic comparison of engineered variants (VK, AVK) versus previously reported hyperactive variants (evo, ee). The Venn diagram shows that while all variants share the critical E229K mutation (except “evo”), VK and AVK possess distinct N-terminal mutations (V5A, D14V) compared to the V74A mutation found in “evo” and “ee.” Relative recombination efficiency of Bxb1 variants in a lysate-based *in vitro* assay using (**B**) 38 bp and (**C**) 30 bp *attB* PCR amplicons. Recombinase inputs were normalized by T2A-linked EGFP fluorescence. Values represent fold-change relative to WT activity. (**D**) PE (PEmax, PE6d) efficiency for 38 bp versus 30 bp *attB* insertion (left panels) and integration efficiency of WT, VK, AVK, evo, and ee recombinases into 38 and 30 bp *attB* sites (PEmax-middle panel, PE6d-right panel) at h*POLR1C* locus. (**E**) PE (PEmax, PE6d, and PE7) efficiency for 38 bp *attB* insertion (left panels) and integration efficiency of WT, VK, AVK, evo, and ee recombinases into 38 bp *attB* sites (right panels) at m*Rosa26* locus in mESCs. Data are represented as the mean of *n* = 3 independent biological replicates (dots); error bars, SEM. *P* values for comparisons to WT were determined by one-way ANOVA. Comparisons not marked with an asterisk are not statistically significant (*P < .05, **P < .01, ***P < .001).

To assess the relative performance of our engineered variants against established high-efficiency recombinases, we performed a head-to-head comparison with the recently reported hyperactive variants, “evoBxb1” (V74A) and “ee” (V74A/E229K/V375I) [29]. A genotypic comparison highlights the distinct mutational profiles resulting from different evolutionary strategies. While the “evo” and “ee” variants share the V74A mutation derived from phage-assisted continuous evolution, our VK (D14V/E229K) and AVK (V5A/D14V/E229K) variants are characterized by N-terminal substitutions (V5A, D14V) identified through our landing pad shortening strategy (Fig. 5A and Supplementary Fig. S5).

To determine whether the improved integration efficiency results from enhanced cellular protein stability or intrinsic enzymatic activity, we established a cell-free *in vitro* recombination assay (Supplementary Fig. S6). We normalized the amount of recombinase in cell lysates using T2A-linked EGFP fluorescence [38] (Supplementary Fig. S6). On the standard 38 bp attB substrate, all engineered variants exhibited robust recombination, demonstrating a substantial increase in activity compared to WT (VK: 5.9-fold, AVK: 6.4-fold, evo: 5.0-fold, ee: 6.4-fold) (Fig. 5B and Supplementary Fig. S6C). Strikingly, on the shortened 30 bp attB substrate, while WT activity was negligible, VK and AVK retained robust catalytic activity. VK and AVK displayed 12.8-fold and 6.8-fold higher efficiency than the WT baseline, respectively. Notably, while the “ee” variant also performed well (8.9-fold), the “evo” variant showed a relatively moderate increase (4.1-fold), indicating a reduced tolerance for the shortened substrate compared to the other variants (Fig. 5C and Supplementary Fig. S6D). This result provides direct evidence that our directed evolution strategy successfully enriched for variants with enhanced intrinsic catalytic efficiency and substrate tolerance, independent of cellular factors.

We next evaluated these variants in a mammalian context by targeting the *POLR1C* locus in HEK293 cells. To assess whether advanced PE platforms could further augment integration efficiency, we performed comparative analyses using PEmax and the improved PE6d system. [39, 40]. While *attB* insertion efficiencies were comparable between PEmax and PE6d, utilizing a 30 bp sequence consistently yielded significantly higher insertion rates (∼18.9%) compared to 38 bp (∼4.4%–5.6%) (Fig. 5D, left panel). Assessment of integration efficiency revealed distinct performance profiles among the recombinases. Despite the increased availability of 30-bp landing pads, the WT Bxb1 shows markedly reduced integration efficiency on these shortened sites (from ∼4.0% on 38 bp to <1.0% on 30 bp) (Fig. 5D, middle, right panel). In stark contrast, the VK, AVK, and “ee” variants effectively leveraged the increased *attB* insertion, resulting in higher overall integration efficiencies on 30 bp sites compared to 38 bp. Notably, the “evo” variant exhibited a distinct profile; consistent with our *in vitro* findings, it showed reduced efficiency on 30 bp sites compared to 38 bp (dropping from ∼4.7% to ∼3.1% with PE6d), failing to fully capitalize on the enhanced PE efficiency (Fig. 5D, middle, right panel). These results confirm that VK and AVK, along with “ee,” are robustly optimized for the compact landing pads generated by PE.

Finally, to assess cross-species applicability, we extended our comparative analysis to mouse cell lines by targeting the *Rosa26* locus. While we confirmed robust activity in mouse NIH 3T3 cells (Supplementary Fig. S7), we sought to validate our variants in a more biologically rigorous model. Common immortalized cell lines such as HEK293 and NIH 3T3 often display extensive aneuploidy, which can confound the precise quantification of editing efficiency and limit their utility for modeling genetic modifications in a therapeutic context [41–43]. To address these limitations, we employed mESCs, a karyotypically normal and pluripotent model highly relevant for transgenic research.

We tested the system using three generations of prime editors: PEmax, PE6d, and the recently developed PE7 [44], to assess integration efficiency across varying *attB* insertion landscapes. In contrast to NIH 3T3 cells, *attB* insertion efficiency in mES cells was relatively low, averaging ~3%, and we did not observe a clear platform-dependent increase in insertion efficiency (PEmax: 3.4%, PE6d: 2.6%, PE7: 2.8%) (Fig. 5E, left panel). However, despite this limited landing pad availability, the performance gap between WT and engineered variants was remarkably pronounced. While WT recombination was minimal (average ∼0.1%), the engineered variants demonstrated substantial integration activity (Fig. 5E, right panel). Specifically, VK and AVK achieved integration rates ranging from 0.5% to 0.9% across conditions, while the “ee” variant reached up to 1.9%. When averaged across all three PE platforms, VK and AVK exhibited an 8.7-fold increase over WT, with “evo” and “ee” showing 7.3-fold and 13.0-fold increases, respectively. The consistent observation of this robust enhancement across all tested platforms, including the latest PE7 system, demonstrates the broad applicability of VK and AVK, confirming their capability to facilitate efficient genome engineering even in challenging cell types such as pluripotent stem cells.

### Engineered Bxb1 variants exhibit high site-specificity with minimal off-target integration

To evaluate the specificity of our engineered Bxb1 recombinase variants and compare their profiles against WT Bxb1 and the previously reported “ee” variant, we performed a comprehensive assessment of their potential for off-target integration at endogenous genomic loci. We selected six candidate off-target sites (OT1–OT6) by referencing pseudo-*attB* sequences for which Bxb1 recombinase-mediated off-target activity was reported in previous *in silico* analyses [33] and whole-genome sequencing studies [13].

**Figure 6.**
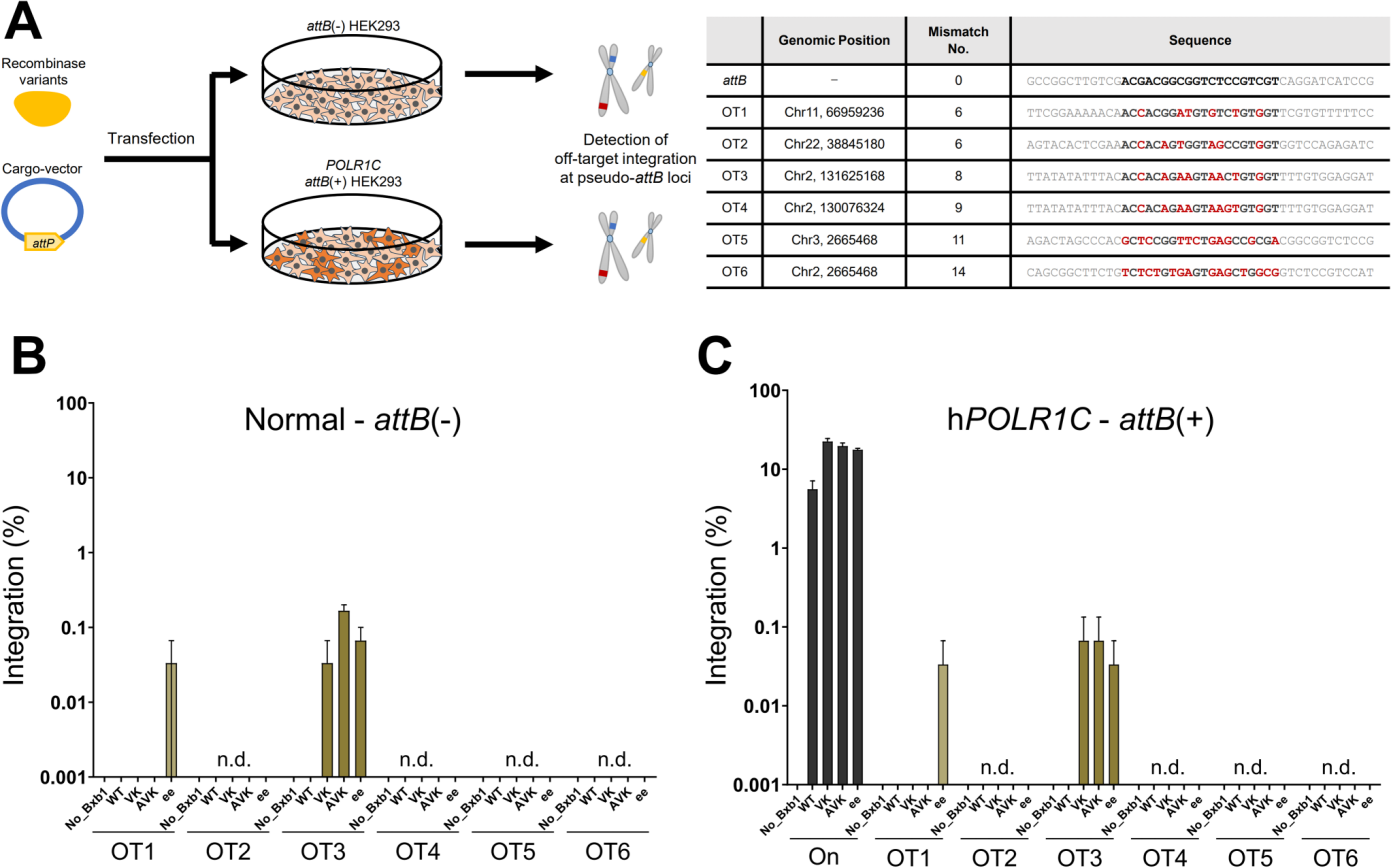
Engineered Bxb1 variants exhibit high site-specificity with minimal off-target integration. (**A**) Overall experimental schematic of the off-target analysis strategy for Bxb1 recombinase variants (left panel) and a table listing the genomic locations and sequences of the six tested pseudo-*attB* sites, designated OT1-OT6 (Off-target site 1–6). (right panel). (**B**) Off-target integration efficiency in normal HEK293 cells (Normal – *attB*(-)). (**C**) On-target and off-target integration efficiency in h*POLR1C* – *attB*(+) cells. Integration efficiency was calculated as a percentage based on three-primer NGS reads (resolution = 0.004%–0.033%). Data are represented as the mean of *n* = 3 independent biological replicates; error bars, SEM. n.d., not detected. *P* values for comparisons to WT were determined by one-way ANOVA. Comparisons not marked with an asterisk are not statistically significant (*P < .05, **P < .01, ***P < .001).

We conducted off-target integration analyses under two distinct experimental conditions. In the first condition, we used normal HEK293 cells with no editing introduced (Normal – *attB*(-)) to assess the background level of off-target activity. In the second condition, we utilized HEK293 cells with an *attB* landing pad inserted at the *POLR1C* locus by PE (h*POLR1C* – *attB*(+)) to reflect conditions similar to the on-target integration experiments. Recombinase variants and the *attP*-containing cargo vector were transfected into each of the two HEK293 cell populations and were harvested and analyzed 96 h later (Fig. 6A). Off-target integration events were then quantified from extracted genomic DNA using targeted next-generation sequencing (NGS).

WT Bxb1 showed no detectable off-target integration at any of the six investigated loci under either experimental condition, aside from its on-target activity at *POLR1C* (5.6%). Among the engineered variants, off-target integration occurred at only two of the six tested sites, and only at very low efficiencies. At off-target site 3 (OT3), integration was detected for VK, AVK, and ee. In normal cells without *attB* insertion (Normal – *attB*(-)), AVK exhibited an average integration efficiency of 0.2%, while VK and ee showed lower efficiencies (below 0.2%) (Fig. 6B). Similarly, in h*POLR1C* (*attB*+) cells, VK, AVK, and ee all displayed minimal off-target effects at OT3, with an average integration efficiency of <0.1% (Fig. 6C). At off-target site 1 (OT1), off-target activity was exclusively observed with the ee variant, showing an average efficiency of <0.04% across all experimental conditions. For all remaining sites (OT2, OT4, OT5, and OT6), no integration was detected for any of the tested variants, including WT, VK, AVK, and ee.

## Discussion

In this study, we report the successful development of highly efficient Bxb1 recombinase variants through a novel directed evolution strategy that uses progressively shortening landing pads as a selective pressure. This approach yielded variants, such as VK and AVK, that exhibit fundamentally enhanced intrinsic activity, demonstrating significantly improved recombination efficiency on standard-length landing pads across diverse genomic loci. Crucially, we demonstrated that this enhanced intrinsic activity allows these variants to overcome the critical bottleneck of PE-mediated landing pad insertion. By synergizing with the high insertion efficiency of short landing pads, our variants dramatically increase the overall efficiency of large-scale gene integration while maintaining high genomic specificity. This establishes our strategy as a robust and efficient platform for advanced genome engineering.

Structural modeling provides further insight into the molecular basis of this enhanced intrinsic activity (Fig. 1E and Supplementary Fig. S5). Analysis based on the AlphaFold3-predicted structure [45] and known serine recombinase domains revealed that the identified mutations are dispersed across major functional regions, likely influencing multiple steps of the recombination pathway [46–50]. Mutations in the N-terminal catalytic domain (NTD; residues 1–150), such as V5A and D14V, may influence initial DNA recognition, while those in the recombinase domain (RD; residues 150–288), including S157G and E229K, likely affect DNA binding and catalysis [50–53]. Furthermore, mutations in the coiled-coil domain (CC; residues 366–432), such as V375M, could modulate the inter-subunit oligomerization required for synaptic complex assembly [54, 55].

Among these substitutions, the E229K mutation in the recombinase domain appears particularly critical. Analysis of the synaptic complex predicted model revealed that the E229K mutation, shared by VK, AVK, and “ee,” substitutes a negatively charged glutamate with a positively charged lysine at the DNA interface (Supplementary Fig. S4). This E229K mutation appears to play a key role in recombination using suboptimal 30 bp *attB* substrates. The substitution likely enhances electrostatic interactions with the DNA backbone, potentially compensating for the reduced stability associated with truncated landing pads. The specificity of this effect is supported by the observation that rational substitutions at nearby residues (E224K, A288K) did not yield additional increases in efficiency (Supplementary Fig. S4). Furthermore, comparative analysis against the “evo” variant (V74A) reveals that while general hyperactivity improves efficiency on standard sites, variants lacking E229K show reduced tolerance for 30 bp substrates (Fig. 5 and Supplementary Fig. S6). In contrast, variants possessing the E229K mutation (“ee,” VK, and AVK) retained robust activity, suggesting that specific adaptations are required to efficiently recognize and catalyze recombination on compact landing pads.

Beyond the fundamental enhancement of the enzyme itself, a pivotal finding of our study is the powerful synergy created when pairing our engineered Bxb1 variants with compact landing pads generated by PE3 (Figs 4 and 5D). This combination provides an elegant solution to a reciprocal bottleneck that has constrained large-scale gene integration: the long landing pads required by WT recombinases are poorly installed by PE, whereas the shorter sequences that are ideal for PE are inefficient substrates for the WT enzyme [13]. Our results illustrate how fundamentally enhancing the efficiency of the recombinase could serve as a breakthrough for this reciprocal bottleneck. While WT Bxb1’s efficiency collapsed on 30 bp sites, completely nullifying the benefit of higher PE efficiency, our VK and AVK variants thrived by efficiently utilizing the more abundant short landing pads, leading to a dramatic increase in overall gene integration. This unique ability to leverage shorter landing pads directly addresses the length-dependent limitations of PE3, offering a powerful and streamlined approach for high-fidelity, large DNA fragment insertion.

The strategy of using short landing pads generally enhances PE efficiency and improves overall integration. However, integration efficiency validation experiments at some genomic loci (*AAVS1, RNF2*) revealed that genomic context remains a critical factor affecting editing efficiency (Supplementary Fig. S8). As observed at the *RNF2* locus, despite the 30 bp *attB* insertion being significantly higher than the 38 bp insertion, subsequent integration efficiency decreased for all recombinases. This discrepancy suggests that locus-specific factors, such as local chromatin accessibility or DNA secondary structure, may restrict recombinase access or compromise conjugate stability, thereby diminishing the benefits typically associated with short landing pads [56, 57]. Notably, even under these challenging conditions, where the WT enzyme showed no detectable integration (0%) at the 30-bp site, our engineered variants retained measurable activity.

The robust performance of our variants was further validated in mESCs, a crucial step to ensure reliability beyond standard immortalized cell lines. We employed karyotypically normal mESCs to validate our findings in a biologically relevant context, avoiding the limitations associated with the aneuploidy of standard cell lines. Notably, the fold-enhancement of the mutant variants compared to the WT was markedly evident in this rigorous setting (with an average increase of ~8.7-fold for AVK), consistent with the pattern observed in NIH 3T3 cells (Fig. 5E and Supplementary Fig. S7). These findings confirm that the superior activity of VK and AVK is not an artifact of transformed cell lines but a robust property maintained in physiological stem cell environments, underscoring their immediate applicability for high-fidelity applications such as the generation of transgenic models and gene therapies.

Our sequence similarity and prediction-based off-target analysis revealed that the engineered Bxb1 variants VK and AVK exhibited only a minimal increase in off-target effects compared to WT Bxb1 (Fig. 6). While WT Bxb1 showed no detectable off-target activity, its on-target efficiency at the *POLR1C* locus was also modest (5.6%). In stark contrast, our engineered variants, despite their substantially enhanced on-target activity, still exhibited minimal off-target integration at only one of the six tested sites (OT3), with frequencies averaging ≤0.2%. Despite these results, however, the potential for off-target effects may have increased as the substrate tolerance of engineered variants expanded. Additional studies, such as unbiased genome-wide off-target analysis [58, 59] or further extended profiling of predicted pseudo-att sites [33, 60], are essential to improve the technical accuracy of recombinase-based genome editing technologies and achieve critical safety enhancements for future clinical applications.

In conclusion, this study establishes a robust strategy for engineering highly efficient site-specific recombinases and provides the resulting variants for advanced genome engineering. The enhanced efficiency of these variants makes them suitable for *in vivo* applications, including the creation of complex transgenic animal models through precise strategies such as recombinase-mediated knock-in and cassette exchange [17, 61–64]. Furthermore, their capacity for efficient, additive insertion of large complementary DNA provides a viable platform for the development of gene therapies for genetic disorders [23, 65, 66]. Beyond the specific variants developed, our work establishes that using landing pad length as a selective pressure is an effective method for isolating globally enhanced recombinases, a strategy that could be utilized to improve other orthogonal recombinase systems, such as φC31 and Pa01. Collectively, our work contributes to improving the efficiency and expanding the applicability of site-specific recombinases for both fundamental research and therapeutic interventions.

## Supplementary Material

gkaf1444_Supplemental_File

## Data Availability

The targeted amplicon sequencing data generated in this study have been deposited in the NCBI Sequence Read Archive (SRA) under the accession number PRJNA1302672. All other relevant data supporting the findings of this study are available from the corresponding author upon reasonable request.
