# Supplementary material for: Directed evolution on compact landing pads yields highly efficient recombinases for large DNA integration
Source: Nucleic Acids Res. 2026 Jan 6;54(1):gkaf1444. doi: 10.1093/nar/gkaf1444 (PMC12774650; doi:10.1093/nar/gkaf1444)
Supplement: gkaf1444_Supplemental_File [file gkaf1444_supplemental_file.pdf]

Supplementary Data

Table S1. Oligonucleotides Used for E. coli-based Directed Evolution

Name	Sequence (5' → 3')	Type	Application
pBAD_*ccdB*_*attP*_52_F	CGCGGTCTCAGTGGTGTACGGTACAAACCCAGTTATGACAACTTGACGGCTACATC	Forward	Construction of pBAD-*attP*-*ccdB*-*attB* vector (shortening of *attP*-*attB* sequence)
pBAD_*ccdB*_*attP*_52_R	TACACCACTGAGACCGCGGTGGTTGACCAGACAAACCACTGTTGGTAACGAATCAGACAATTGACGGCTTGA	Reverse	
pBAD_*ccdB*_*attB*_44_F	GTCTCCGTCGTCAGGATCATCCGTAAGCTGTTTTGGCGGATGAGAGAAGAT	Forward	
pBAD_*ccdB*_*attB*_44_R	TGATCCTGACGACGGAGACCGCCGTCGTCGACAAGCCGGCGTTAAGAGCCTATTCCCCAGAACATCA	Reverse	
pBAD_*ccdB*_*attP*_46_F	CGCGGTCTCAGTGGTGTACGGTACAAACGTTATGACAACTTGACGGCTACATC	Forward	
pBAD_*ccdB*_*attP*_46_R	TACACCACTGAGACCGCGGTGGTTGACCAGACAAACTGTTGGTAACGAATCAGACAATTGACGGCTTGA	Reverse	
pBAD_*ccdB*_*attB*_36_F	GTCTCCGTCGTCAGGATCATAAGCTGTTTTGGCGGATGAGAGAAGAT	Forward	
pBAD_*ccdB*_*attB*_36_R	TGATCCTGACGACGGAGACCGCCGTCGTCGACAAGCGTTAAGAGCCTATTCCCCAGAACATCA	Reverse	
pBAD_*ccdB*_*attB*_34_F	CTTAACCTTGTCGACGACGGCGGTCTCCGTCGTCAGGATCTAAGCTGTTTTGGCGGATGAGAGAAGAT	Forward	
pBAD_*ccdB*_*attB*_34_R	CCGTCGTCGACAAGGTTAAGAGCCTATTCCCCAGAACATCA	Reverse	
pBAD_*ccdB*_*attB*_33_F	GGTCTCCGTCGTCAGGATTAAGCTGTTTTGGCGGATGAGAGAAGAT	Forward	
pBAD_*ccdB*_*attB*_33_R	TAATCCTGACGACGGAGACCGCCGTCGTCGACAAGTTAAGAGCCTATTCCCCAGAACATCA	Reverse	
pBAD_*ccdB*_*attB*_32_F	TAATTGTCGACGACGGCGGTCTCCGTCGTCAGGATAAGCTGTTTTGGCGGATGAAGAAGAT	Forward	
pBAD_*ccdB*_*attB*_32_R	CGCCGTCGTCGACAATTAAGAGCCTATTCCCCAGAACATCA	Reverse	
pBAD_*ccdB*_*attB*_30_F	TAACTGTCGACGACGGCGGTCTCCGTCGTCAGGAAAGCTGTTTTGGCGGATGAAGAAGAT	Forward	
pBAD_*ccdB*_*attB*_30_R	CGCCGTCGTCGACAGTTAAGAGCCTATTCCCCAGAACATCA	Reverse	
pBAD_*ccdB*_*attB*_28_F	GTCTCCGTCGTCAGGTAAGCTGTTTTGGCGGATGAGAGAAGAT	Forward	
pBAD_*ccdB*_*attB*_28_R	GCTTACCTGACGACGGAGACCGCCGTCGTCGACGTTAAGAGCCTATTCCCCAGAACATCA	Reverse	
pLtetO_*ccdB*_Random_F	GAAAAGAATTCAAAAGATCTAAAGAGGACTTCGGATCTATG	Forward	Error-prone PCR for Bxb1 library construction from pLtetO-Bxb1 vector
pLtetO_*ccdB*_Random_R	ATTTGATGCCTGGAGATCCTTACTCG	Reverse	

Table S2. Target Loci and Guide RNA Sequences

Target	Type	Target Sequence (5' → 3')	RTT	PBS
h*CCR5*	pegRNA_A	AAGTGTGATCACTTGGGTGG	ATGATCCTGACGACGGAGACCGCCGTCGT CGACAAGCCGG	CCCAAGTGATC
h*CCR5*	pegRNA_B	GTATGGAAAATGAGAGCTGC	GGCTTGTCGACGACGGCGGTCTCCGTCGT CAGGATCAT	GCTCTCATTTTC
h*HEK3*	pegRNA_A	GCCCAGACTGAGCACGTGA	ATGATCCTGACGACGGAGACCGCCGTCGT CGACAAGCCGG	CGTGCTCAGTCTG
h*HEK3*	pegRNA_B	TCAACCAGTATCCCGGTGC	GGCTTGTCGACGACGGCGGTCTCCGTCGT CAGGATCATCCG	GGATACTGG
h*AAVS1*	pegRNA_A	CAGAGCCAGGAACCCCTGT	ATGATCCTGACGACGGAGACCGCCGTCGT CGACAAGCCGG	GGTTCCTGGCT
h*AAVS1*	pegRNA_B	TCCTTGGCAAGCCCAGGAG	GGCTTGTCGACGACGGCGGTCTCCGTCGT CAGGATCATCCG	CTGGGCTTGCCAA
h*POLR1C*	pegRNA_A	ATGTCTGCAGGCCAGATGA	ACGACGGAGACCGCCGTCGTCGACAAGCC	TCTGGCCT
h*POLR1C*	pegRNA_B	ATGTACAGAGAGCCCAGGGC	ACGACGGCGGTCTCCGTCGTCAGGATCAT	CTGGGCTC
h*RNF2*	pegRNA_A	TCATCTTAGTCATTACCTG	ATGATCCTGACGACGGAGACCGCCGTCGT CGACAAGCCGG	TAATGACTAAGATG
h*RNF2*	pegRNA_B	TCTCAGGCTGTGCAGACAAA	GGCTTGTCGACGACGGCGGTCTCCGTCGT CAGGATCATCCG	TCTGCACAGCC
h*RUNX1*	pegRNA_A	AGTCCCCCGCCTTCAGAAG	ACGACGGAGACCGCCGTCGTCGACAAGCC	CTGAAGGC
h*RUNX1*	pegRNA_B	ATGAAGCACTGTGGGTACGA	ACGACGGCGGTCTCCGTCGTCAGGATCAT	TACCCACAG
h*ACTB*	pegRNA – 38 bp	GCTATTCTCGCAGCTCACCA	GACGAGCGCGGCGATATCATCATCCATGG ATGATCCTGACGACGGAGACCGCCGTCGT CGACAAGCC	TGAGCTGCGAGAA
h*ACTB*	pegRNA – 30 bp	GCTATTCTCGCAGCTCACCA	GACGAGCGCGGCGATATCATCATCCATGG TCCTGACGACGGAGACCGCCGTCGTCGAC A	TGAGCTGCGAGAA
h*ACTB*	Nicking sgRNA	GAAGCCGGCCTTGCACATGC	-	-
h*LNMB1*	pegRNA – 38 bp	CTGTCTCCGCCGCCCGCCA	CGGGGGTCGCAGTCGCCATGATGATCCTG ACGACGGAGACCGCCGTCGTCGACAAGCC	CGGGCGGCG
h*LNMB1*	pegRNA – 30 bp	CTGTCTCCGCCGCCCGCCA	CGGGGGTCGCAGTCGCCATGTCCTGACGA CGGAGACCGCCGTCGTCGACA	CGGGCGGCG
h*LNMB1*	Nicking sgRNA	ACAGGCGCGTGGGGCTCAG	-	-
h*NOLC1*	pegRNA – 38 bp	CGTATTGCCTGGAGGATGG	GAACCACGCGGCGAATGCCGGCGTCCGC CCATGATCCTGACGACGGAGACCGCCGTC GTCGACAAGCC	TCCTCCAGGCAAT
h*NOLC1*	pegRNA – 30 bp	CGTATTGCCTGGAGGATGG	GAACCACGCGGCGAATGCCGGCGTCCGC CTCCTGACGACGGAGACCGCCGTCGTCGA CA	TCCTCCAGGCAAT
h*NOLC1*	Nicking sgRNA	AGCCGAGCACGAGGGGATAC	-	-
m*Rosa26*	pegRNA_A	TCTACTGTTCACTCTAACAG	ACGACGGAGACCGCCGTCGTCGACAAGCC	TTAGAGTGAA
m*Rosa26*	pegRNA_B	TAATCTGCTAGTATATCCGT	ACGACGGCGGTCTCCGTCGTCAGGATCAT	GATATACTAG
h*POLR1C*	pegRNA – 38 bp	ATGTCTGCAGGCCAGATGA	ATCTGGAGCCCTCAATGATCCTGACGACG GAGACCGCCGTCGTCGACAAGCC	TCTGGCCT
h*POLR1C*	pegRNA – 30 bp	ATGTCTGCAGGCCAGATGA	ATCTGGAGCCCTCATCCTGACGACGGAGA CCGCCGTCGTCGACA	TCTGGCCT
h*POLR1C*	Nicking sgRNA	ATGTACAGAGAGCCCAGGGC	-	-
h*RNF2*	pegRNA – 38 bp	TCATCTTAGTCATTACCTG	GTTACAACGAACACCTCAGATGATCCTGAC GACGGAGACCGCCGTCGTCGACAAGCC	GTAATGACTAAGATG
h*RNF2*	pegRNA – 30 bp	TCATCTTAGTCATTACCTG	GTTACAACGAACACCTCAGTCCTGACGAC GGAGACCGCCGTCGTCGACA	GTAATGACTAAGATG
h*RNF2*	Nicking sgRNA	TCAACCATTAAGCAAAACAT	-	-
h*AAVS1*	pegRNA – 38 bp	CAGAGCCAGGAACCCCTGT	CCTTCCCTACAATGATCCTGACGACGGAG ACCGCCGTCGTCGACAAGCC	GGGGTTCCTGGCT
h*AAVS1*	pegRNA – 30 bp	CAGAGCCAGGAACCCCTGT	CCTTCCCTACATCCTGACGACGGAGACCG CCGTCGTCGACA	GGGGTTCCTGGCT
h*AAVS1*	Nicking sgRNA	TCCTTGGCAAGCCCAGGAG	-	-

Table S3. Oligonucleotides Used for Mammalian Cell Expression Vector Construction

Name	Sequence (5’-->3’)	Type	Application
pCMV_Bxb1_V5A_F	ATAGGGAGAGCCGCCACCATGAGAGCCCTGGCCGTGATTAGACTGA GCCGAGTGAC	Forward	Site-specific mutagenesis for Bxb1 variant cloning
pCMV_Bxb1_V5A_R	GGTGGCGGCTCTCCCTAT	Reverse	
pCMV_Bxb1_D14V_F	CGTGATTAGACTGAGCCGAGTGACAGTTGCCACCACAAGTCCCGAG AG	Forward	
pCMV_Bxb1_D14V_R	ACTCGGCTCAGTCTAATCACG	Reverse	
pCMV_Bxb1_V74A_F	CTTTCGAGGAACAGCCCTTTGATGCCATCGTCGCCTACAGAGTGGA	Forward	
pCMV_Bxb1_V74A_R	AAAGGGCTGTTCCTCGAAAG	Reverse	
pCMV_Bxb1_A77V_F	ACAGCCCTTTGATGTGATCGTCGTTTACAGAGTGGACCGGCTGAC	Forward	
pCMV_Bxb1_A77V_R	CGATCACATCAAAGGGCTGT	Reverse	
pCMV_Bxb1_I87L_F	CCGCTCACTCCGACATCTCCAGCAGCTGGTGCATTGG	Forward	
pCMV_Bxb1_I87L_R	CTGGAGATGTCGGAGTGAGCGGGTCAGCCGGTCCACTCTGTA	Reverse	
pCMV_Bxb1_H111L_F	TACTACCACACCTTTTGCCG	Forward	
pCMV_Bxb1_H111L_R	CGGCAAAAGGTGTGGTAGTATCGAACAGGGCTTCTGTTGCGCTGAC CAC	Reverse	
pCMV_Bxb1_S157G_F	TAGGGCCGGTAAATACAGAGGCGGGCTGCCCCCTTGGGGATATCTC CCTACCA	Forward	
pCMV_Bxb1_S157G_R	CCTCTGTATTTACCGGCCCTA	Reverse	
pCMV_VK_E224K_F	TTATTTTGCTCAGCTGCAGGGAAGAAAGCCACAGGGAAGAAAATGGA G	Forward	Site-specific mutagenesis for VK+E224K variant cloning
pCMV_VK_E224K_R	CCTGCAGCTGAGCAAAATAA	Reverse	
pCMV_Bxb1_E229K_F	TTATTTTGCTCAGCTGCAGGGAAGAGAGCCACAGGGAAGAAAATGGA GTGCTACTGCACTGAAGA	Forward	Site-specific mutagenesis for Bxb1 variant cloning
pCMV_Bxb1_E229K_R	CCTGCAGCTGAGCAAAATAA	Reverse	
pCMV_VK_A288K_F	AAGAAACCTGCCGTGAGCACCCCAAGCCTGCTCCTGAGGGTGCTGT TCT	Forward	Site-specific mutagenesis for VK+A288K variant cloning
pCMV_VK_A288K_R	GGTGCTCACGGCAGGTTTCTTGCGACTAGTCTTGACCAGTTCTGC	Reverse	
pCMV_Bxb1_V375I_F	AAAAGTGTGGGTGGCAGGATCTGACTCCGCTATCGAGCTGGCAGAA GTCAATGC	Forward	Site-specific mutagenesis for Bxb1 variant cloning
pCMV_Bxb1_V375I_R	AGATCCTGCCACCCACACT	Reverse	
pCMV_Bxb1_V375M_F	AGTGTGGGTGGCAGGATCTGACTCCGCTATGGAGCTGGCAGAAGTC AAT	Forward	
pCMV_Bxb1_V375M_R	AGATCCTGCCACCCACACT	Reverse	
pCMV_Bxb1_A411T_F	GCTCTGGACGCACGAATTACAGCACTCGCTGCTAGACAGGAGGAAC	Forward	
pCMV_Bxb1_A411T_R	AATTCGTGCGTCCAGAGC	Reverse	
pCMV_Bxb1_W432L_F	AACTGGAGGGCCTGGAGGCCAGGCCCTCTGGATGGGAGCTGCGAG AAACCGGACAGAGGTTTG	Forward	
pCMV_Bxb1_W432L_R	CCTCCAGGCCCTCCAGTT	Reverse	

Table S4. Oligonucleotides Used for Targeted Amplicon Sequencing (NGS) and Off-target Analysis

Name	Target gene	Sequence (5' → 3')	Type	Application
h*CCR5*_DF	h*CCR5*	GTGACTGGAGTTCAGACGTGTGCTCTTCCGATCTGCAGATGACCATGACAAGCA	Adapter Forward	TwinPE insertion NGS / recombinase integration three primer NGS
h*CCR5*_DR	h*CCR5*	GTGACTGGAGTTCAGACGTGTGCTCTTCCGATCTGCAGATGACCATGACAAGCA	Adapter Reverse	
h*HEK3*_DF	h*HEK3*	ACACTCTTTCCCTACACGACGCTCTTCCGATCTGCATGCATTTGTAGGCTTGA	Adapter Forward	
h*HEK3*_DR	h*HEK3*	GTGACTGGAGTTCAGACGTGTGCTCTTCCGATCTAGGGACCTCCCTAGGTGCT	Adapter Reverse	
h*AAVS1*_DF	h*AAVS1*	ACACTCTTTCCCTACACGACGCTCTTCCGATCTGGTCCAGGCCAAGTAGGTG	Adapter Forward	
h*AAVS1*_DR	h*AAVS1*	GTGACTGGAGTTCAGACGTGTGCTCTTCCGATCTCTTCCCTAAGGCCCTGCTCT	Adapter Reverse	
h*POLR1C*_DF	h*POLR1C*	ACACTCTTTCCCTACACGACGCTCTTCCGATCTTTGGTGCCAAATTCTTCTCC	Adapter Forward	
h*POLR1C*_DR	h*POLR1C*	GTGACTGGAGTTCAGACGTGTGCTCTTCCGATCTTTTCTGACCTCCCAAACAGC	Adapter Reverse	
h*RNF2*_DF	h*RNF2*	ACACTCTTTCCCTACACGACGCTCTTCCGATCTTTCATGTTCTAAAAATGTATCCCAGT	Adapter Forward	
h*RNF2*_DR	h*RNF2*	GTGACTGGAGTTCAGACGTGTGCTCTTCCGATCTCCATAGCACTTCCCTTCCAA	Adapter Reverse	
h*RUNX1*_DF	h*RUNX1*	ACACTCTTTCCCTACACGACGCTCTTCCGATCTTGAGGCTGAAACAGTGACCT	Adapter Forward	
h*RUNX1*_DR	h*RUNX1*	GTGACTGGAGTTCAGACGTGTGCTCTTCCGATCTGGTGAAACAAGCTGCCATTT	Adapter Reverse	
h*ACTB*_DF	h*ACTB*	ACACTCTTTCCCTACACGACGCTCTTCCGATCTCGACCTCGGCTCACAGCG	Adapter Forward	PE3 insertion NGS / recombinase integration three primer NGS
h*ACTB*_DR	h*ACTB*	GTGACTGGAGTTCAGACGTGTGCTCTTCCGATCTCAGCCAGCTCCCCTACCT	Adapter Reverse	
h*NOLC1*_DF	h*NOLC1*	ACACTCTTTCCCTACACGACGCTCTTCCGATCTACTGCCCCTGTTGGGTTC	Adapter Forward	
h*NOLC1*_DR	h*NOLC1*	GTGACTGGAGTTCAGACGTGTGCTCTTCCGATCTCCTGAAGCCTTGTGGTCATC	Adapter Reverse	
h*LNMB1*_DF	h*LNMB1*	ACACTCTTTCCCTACACGACGCTCTTCCGATCTGCCGTCCCCTCCTTATCAC	Adapter Forward	
h*LNMB1*_DR	h*LNMB1*	GTGACTGGAGTTCAGACGTGTGCTCTTCCGATCTGCGCACCTTGTCGATGTA	Adapter Reverse	
m*Rosa26*_DF	m*Rosa26*	ACACTCTTTCCCTACACGACGCTCTTCCGATCTACTCCACTGGGTGGATTTTTA	Adapter Forward	TwinPE insertion NGS / recombinase integration three primer NGS
m*Rosa26*_DR	m*Rosa26*	GTGACTGGAGTTCAGACGTGTGCTCTTCCGATCTTCAGAACAAGGTAGTATAAAGCTGGTA	Adapter Reverse	
*attB*_OT1_DF	Pseudo_*attB*_OT1	ACACTCTTTCCCTACACGACGCTCTTCCGATCTGCCTACTTGCCCTTCTTCCT	Adapter Forward	Recombinase integration three primer NGS
*attB*_OT1_DR	Pseudo_*attB*_OT1	GTGACTGGAGTTCAGACGTGTGCTCTTCCGATCTGGAAAGCTTTGACAAGTGGAA	Adapter Reverse	
*attB*_OT2_DF	Pseudo_*attB*_OT2	ACACTCTTTCCCTACACGACGCTCTTCCGATCTCTGGGAACACTGGACAAAATCC	Adapter Forward	
*attB*_OT2_DR	Pseudo_*attB*_OT2	GTGACTGGAGTTCAGACGTGTGCTCTTCCGATCTGCTGTGGTTATTCCCAGCTC	Adapter Reverse	
*attB*_OT3_DF	Pseudo_*attB*_OT3	ACACTCTTTCCCTACACGACGCTCTTCCGATCTGGAAATAAGTTATCACAATGGGAAAT	Adapter Forward	
*attB*_OT3_DR	Pseudo_*attB*_OT3	GTGACTGGAGTTCAGACGTGTGCTCTTCCGATCTTCGCGATTCTTAAAAGGAGAGG	Adapter Reverse	
*attB*_OT4_DF	Pseudo_*attB*_OT4	ACACTCTTTCCCTACACGACGCTCTTCCGATCTCGCAATGGCAAATAATCTCA	Adapter Forward	
*attB*_OT4_DR	Pseudo_*attB*_OT4	GTGACTGGAGTTCAGACGTGTGCTCTTCCGATCTCGCGATTCTTAAAAGGAGAGG	Adapter Reverse	
*attB*_OT5_DF	Pseudo_*attB*_OT5	ACACTCTTTCCCTACACGACGCTCTTCCGATCTTCCTCCATCTGTGAGTCAGC	Adapter Forward	
*attB*_OT5_DR	Pseudo_*attB*_OT5	GTGACTGGAGTTCAGACGTGTGCTCTTCCGATCTAGACAGAAGCCCTCGCTCAC	Adapter Reverse	
*attB*_OT6_DF	Pseudo_*attB*_OT6	ACACTCTTTCCCTACACGACGCTCTTCCGATCTAAGTTATCACAATGACAAATAATCTCA	Adapter Forward	
*attB*_OT6_DR	Pseudo_*attB*_OT6	GTGACTGGAGTTCAGACGTGTGCTCTTCCGATCTCGTGATTCTTAAAAGGAGAAGTCA	Adapter Reverse	
pCargo_DF	pCargo_vector	ACACTCTTTCCCTACACGACGCTCTTCCGATCTACATGGTCCTGCTGGAGTTC	Adapter Forward	
pCargo_DR	pCargo_vector	GTGACTGGAGTTCAGACGTGTGCTCTTCCGATCTTCAGGTTCAGGGGGAGGT	Adapter Reverse	

Table S5. Mutation Frequency of Surviving Clones from Directed Evolution Rounds 6 and 7

Clone ID	Mutation	Round 6 – Frequency	Round 7 – Frequency
Mutant 1	V5A/A77V/S157G/A411T	31 (62%)	0 (0%)
Mutant 2	D14V/V375M/W432L	7 (14%)	1 (5%)
Mutant 3	E229K	2 (4%)	1 (5%)
Mutant 4	H111L	10 (20%)	18 (90%)
Total		50	20

Supplementary Figures

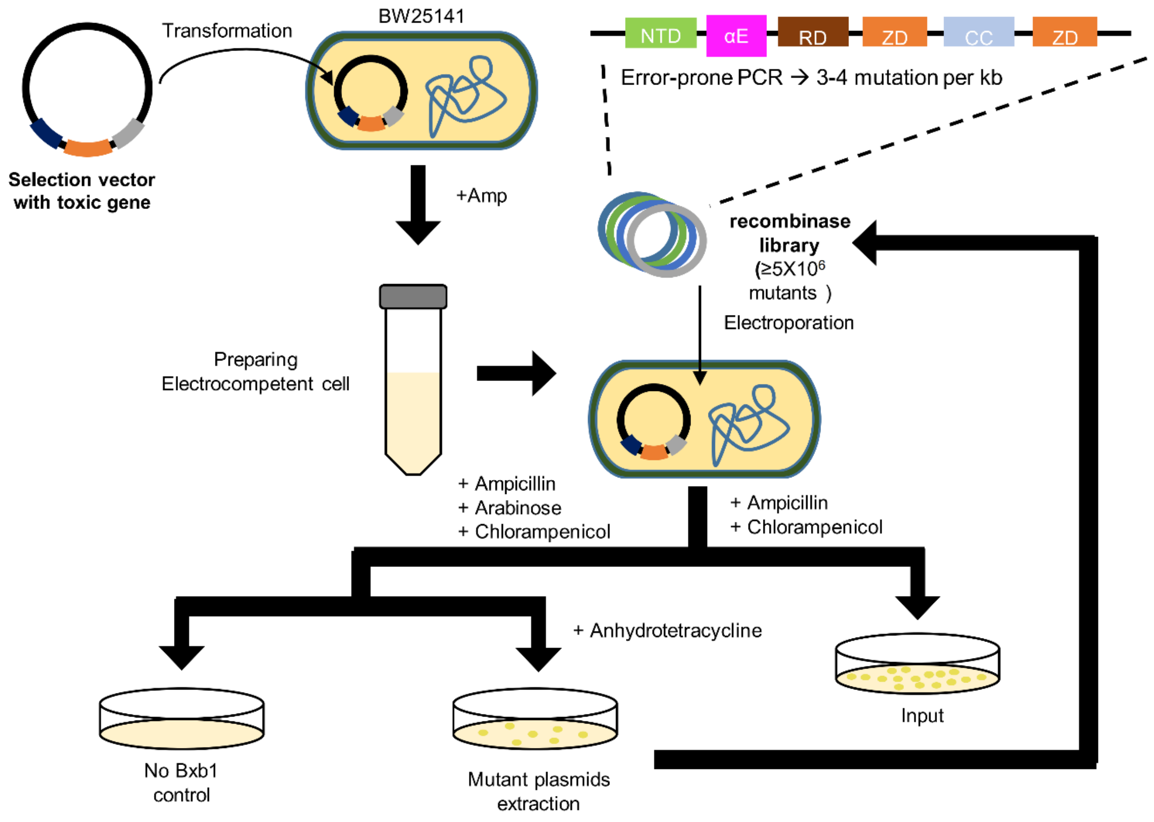

Figure S1. Detailed schematic of the E. coli-based directed evolution system for the enrichment of efficient recombinase mutants active on shortened landing pads. BW25141 E. coli host cells are first transformed with a selection vector containing a toxic *ccdB* gene flanked by *attP* and *attB* sites. A Bxb1 recombinase mutant library, generated by error-prone PCR, is then introduced via electroporation. The selection relies on inducing *ccdB* expression with L-arabinose and recombinase expression with anhydrotetracycline (aTc). Two control plates are used to assess selection efficiency. One control plate (ampicillin/chloramphenicol only) is used to quantify the total input colony count, while the other (L-arabinose/ampicillin/chloramphenicol, no aTc) confirms the lethality of *ccdB* without recombinase induction. Only E. coli colonies with an active recombinase (i.e., capable

of excising the *ccdB* gene) survive on plates containing both L-arabinose and aTc. Plasmids from these enriched colonies are recovered and used for the next selection round, where they are introduced again into cells with selection vectors having progressively shorter *attP*-*attB* lengths. This iterative process enriches for mutant recombinases optimized for shortened *attB* sequences.

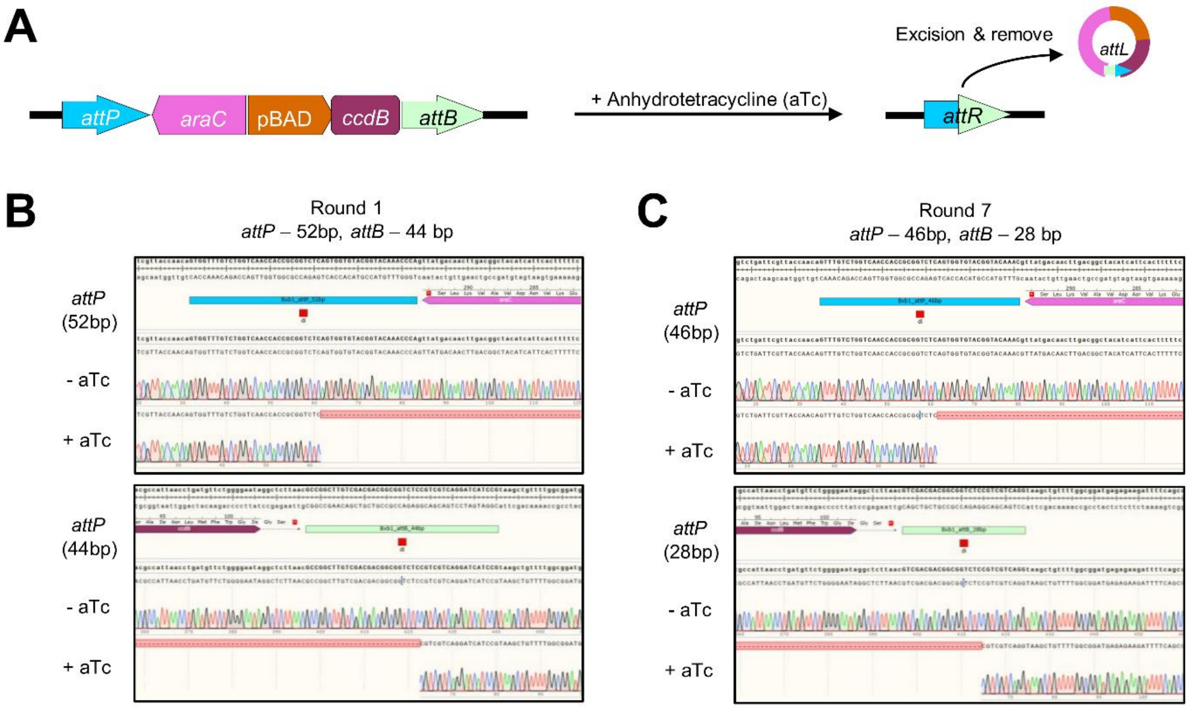

Figure S2. Sequence validation of *ccdB* cassette excision. (A) A schematic diagram of *ccdB* gene excision and *attR* formation within the selection vector. The structure of the *attP*-araC-pBAD-*ccdB*-*attB* selection vector is illustrated, depicting how the toxic *ccdB* gene cassette is precisely excised upon anhydrotetracycline (aTc) induction, resulting in the formation of the *attR* recombination product. **(B)** Sanger sequencing alignment data from Round 1 (*attP*-52bp, *attB*-44bp) with and without aTc induction. **(C)** Sanger sequencing alignment data from Round 7 (*attP*-46bp, *attB*-28bp) with and without aTc induction.

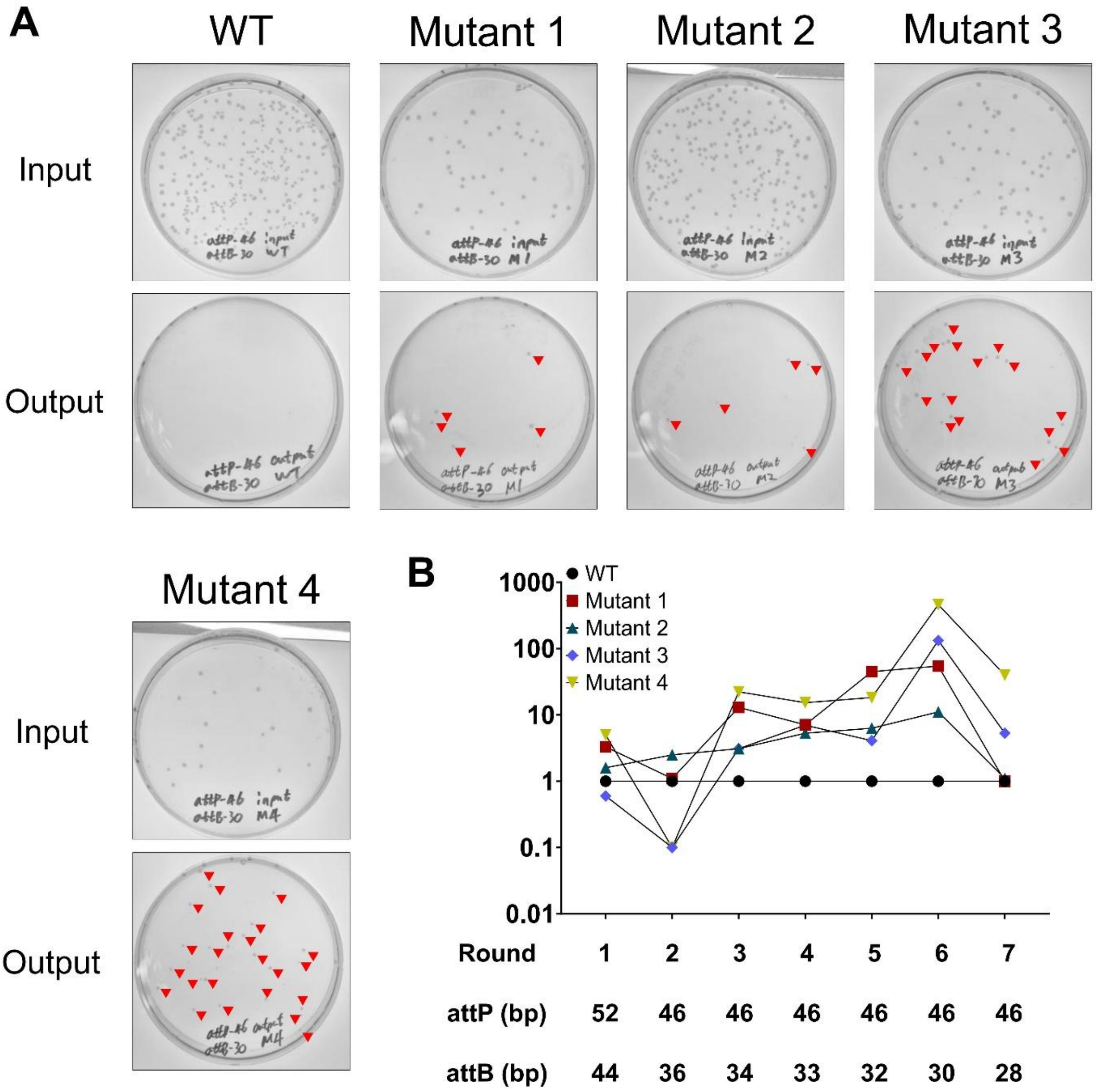

Figure S3. Analysis of colony growth dynamics of bxb1 variants under selective pressure. (A) Representative images of bacterial colonies surviving on selective media plate. E. coli cells expressing Wild-type (WT) or Mutants 1–4 were plated on selective media containing arabinose and aTc with a shortened 30 bp attB selection vector. **(B)** Normalized survival ratios of the four representative mutants across selection rounds 1–7. Survival rates were measured individually for each mutant and normalized to the survival rate of WT Bxb1 under identical selection conditions.

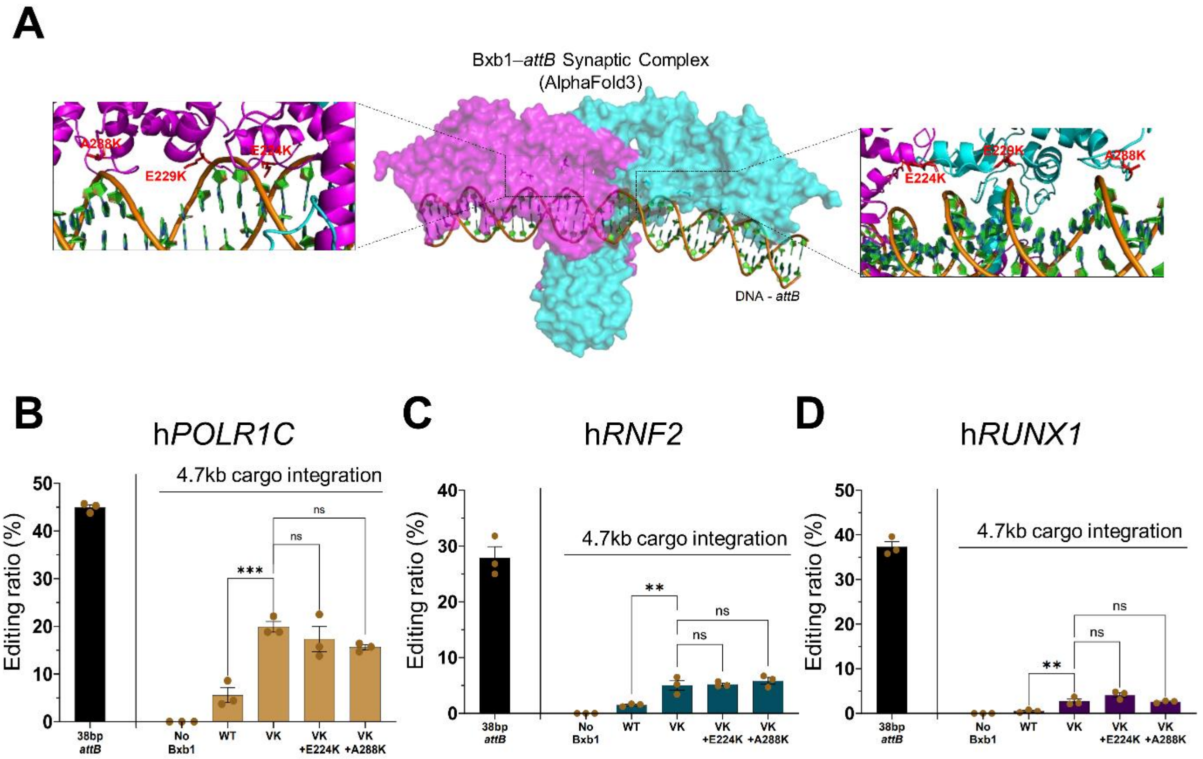

Figure S4. Evaluation of additional rational mutations based on predicted protein structure. (A) Predicted structure of the Bxb1–*attB* synaptic complex, highlighting the positions of E229 and other rationally selected candidate residues (E224, A288) for mutation. **(B-D)** *AttB* insertion and subsequent integration efficiencies of Bxb1 variants at diverse genomic loci. Data are represented as the mean of n=3 independent biological replicates (dots); error bars, SEM. P values for comparisons to WT were determined by one-way ANOVA. Comparisons not marked with an asterisk are not statistically significant (*P < 0.05, **P < 0.01, ***P < 0.001).

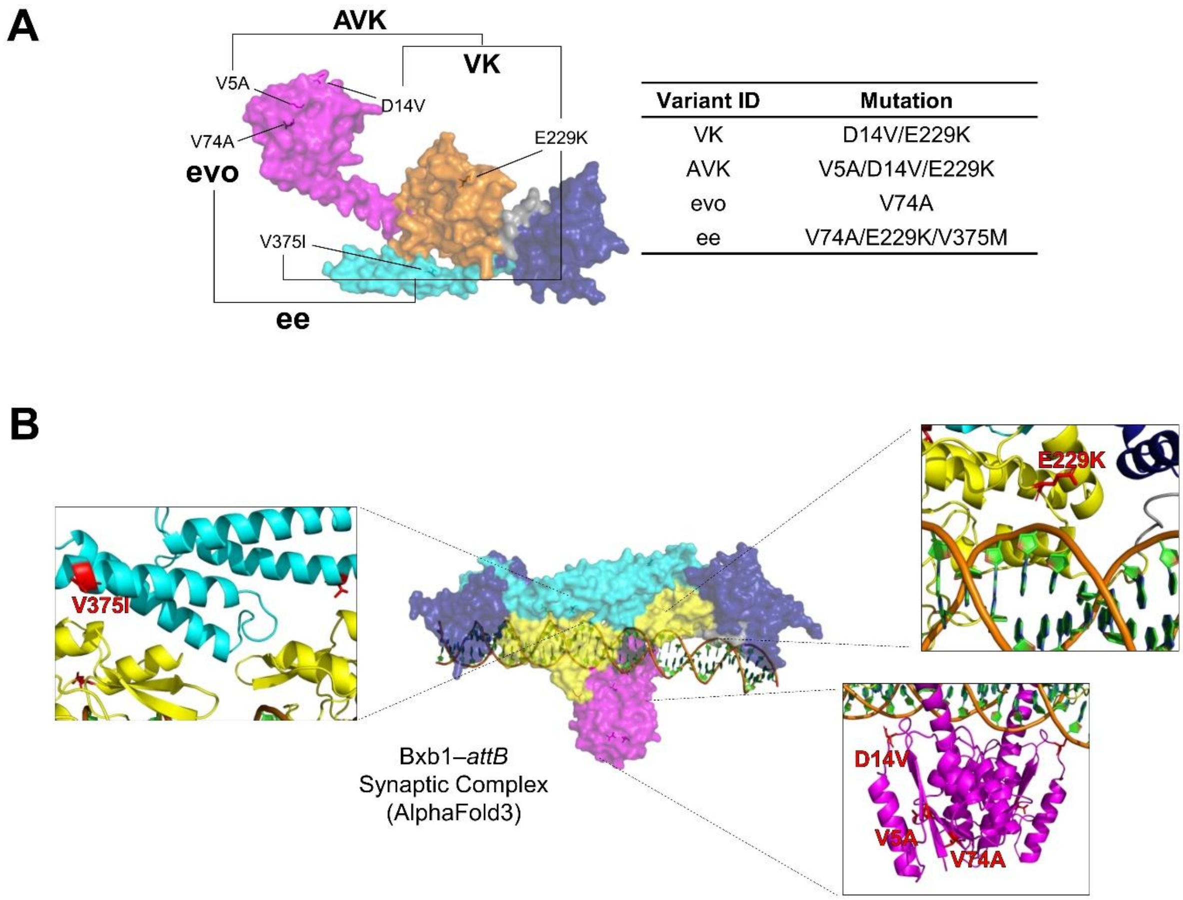

Variant ID	Mutation
VK	D14V/E229K
AVK	V5A/D14V/E229K
evo	V74A
ee	V74A/E229K/V375M

Figure S5. Comparative analysis between the engineered Bxb1 variants developed in this study and previously reported hyperactive Bxb1 variants. (A) Structural comparison of engineered variants (VK, AVK) versus previously reported variants (evo, ee) in predicted Bxb1 monomer structure. The location of mutations is mapped onto the Bxb1 dimer structure. **(B)** AlphaFold3 prediction of the Bxb1 (dimer)-DNA (*attB*) synaptic complex. Unique mutations for VK/AVK (V5A, D14V) and evo/ee (V74A, V375I) are highlighted, alongside the shared E229K mutation.

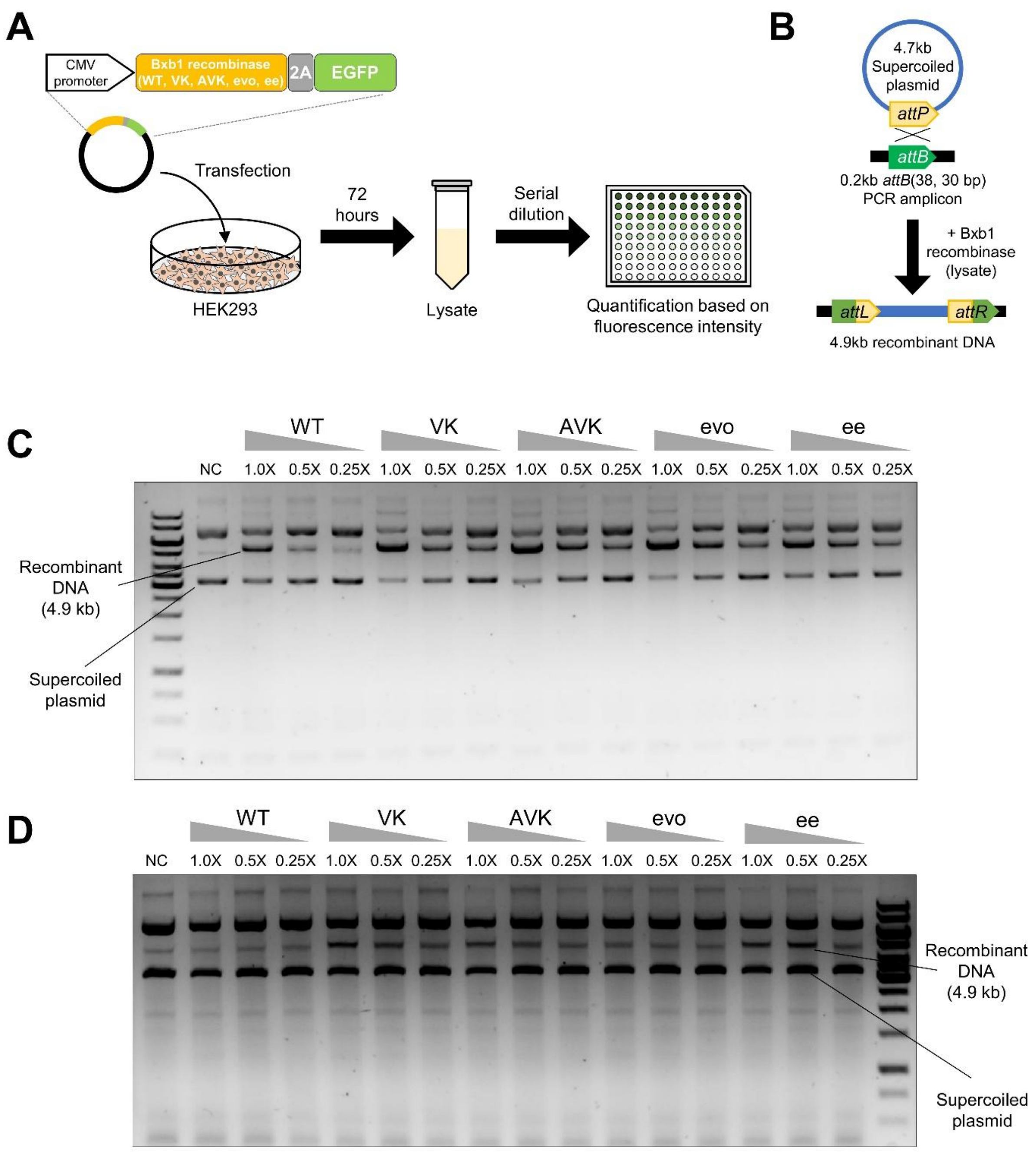

Figure S6. Assessment of the intrinsic enzymatic activity of Bxb1 recombinase variants through a cell-lysate-based in vitro recombination assay. (A) Schematic workflow for lysate preparation and quantification. HEK293 cells were transfected with plasmids encoding recombinase variants (WT, VK, AVK, evo, ee) linked to EGFP via a T2A peptide. After 72 hours, cell lysates were harvested, and the relative concentration of recombinase was normalized based on EGFP fluorescence intensity

to ensure equal protein input for the assay. **(B)** Schematic representation of the in vitro recombination reaction. A supercoiled plasmid containing an *attP* site (4.7 kb) was recombinated with a linear PCR amplicon containing the *attB* site (~0.2 kb) by Bxb1 recombinases in cell lysate. Successful recombination results in a linearized 4.9 kb product. **(C, D)** Representative agarose gel images of recombination products using (C) 38 bp and (D) 30 bp attB substrates.

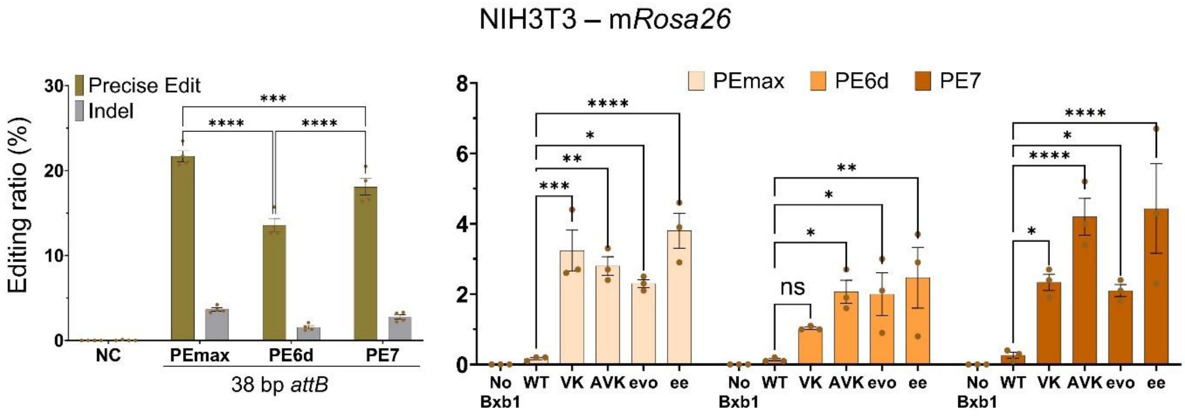

Figure S7. A comparative evaluation of Bxb1 recombinase variants in mouse NIH 3T3 cells. Integration efficiency of Bxb1 variants at the m*Rosa26* locus in NIH 3T3 cells using PEmax, PE6d, and PE7 editors. Data are represented as the mean of n=3 independent biological replicates (dots); error bars, SEM. P values for comparisons to WT were determined by one-way ANOVA. Comparisons not marked with an asterisk are not statistically significant (*P < 0.05, **P < 0.01, ***P < 0.001).

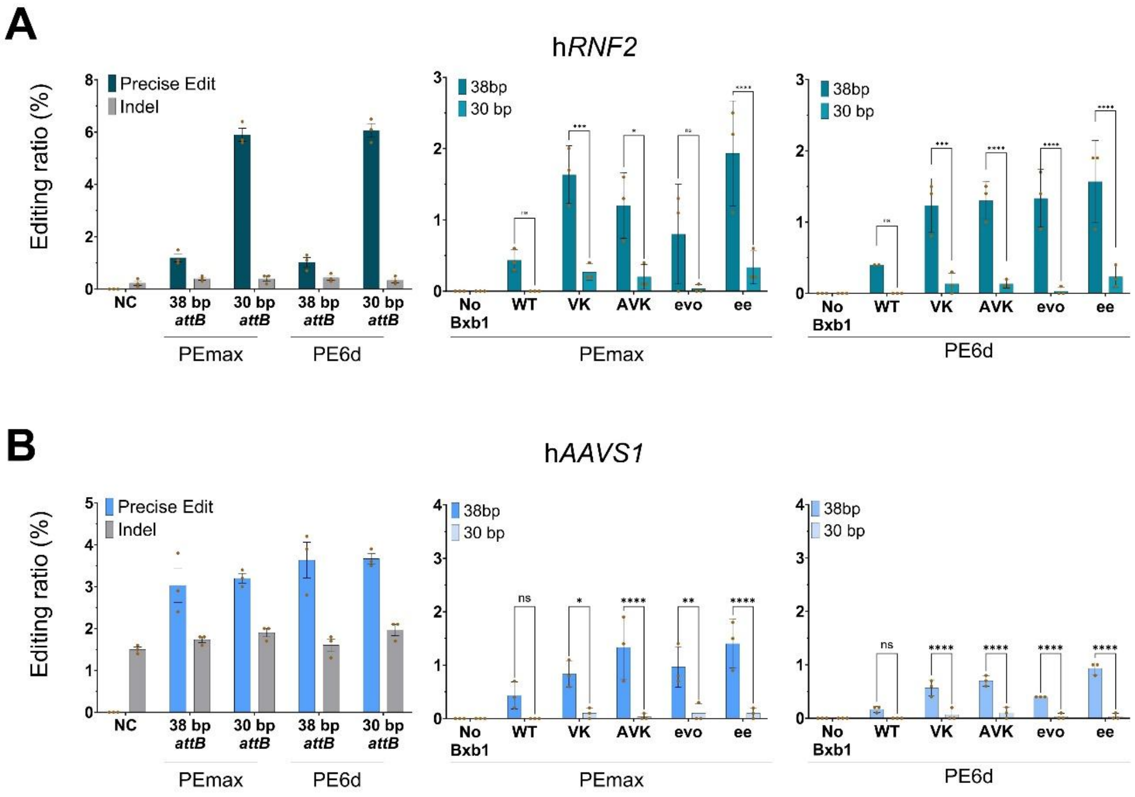

Figure S8. Locus-specific integration efficiencies of Bxb1 recombinase variants reveal constraints imposed by chromatin-dependent accessibility. Comparison of PE3-mediated *attB* insertion and subsequent integration efficiency at the h*RNF2* (A) and h*AAVS1* (B) loci using PEmax and PE6d. Data are represented as the mean of n=3 independent biological replicates (dots); error bars, SEM. P values for comparisons to WT were determined by one-way ANOVA. Comparisons not marked with an asterisk are not statistically significant (*P < 0.05, **P < 0.01, ***P < 0.001).
